# Regioselectivity of oxidation by a polysaccharide monooxygenase from *Chaetomium thermophilum*

**DOI:** 10.1186/s13068-018-1156-2

**Published:** 2018-06-05

**Authors:** Chen Chen, Jinyin Chen, Zhigang Geng, Meixia Wang, Ning Liu, Duochuan Li

**Affiliations:** Department of Mycology, Shandong Agricultural University, Taian, 271018 Shandong China

**Keywords:** *Chaetomium thermophilum*, Auxiliary activity family 9 (AA9), Polysaccharide monooxygenase (PMO), Regioselectivity of oxidation, C1, C4 and C6 oxidation

## Abstract

**Background:**

Polysaccharide monooxygenases (PMOs) of the auxiliary activity 9 (AA9) family have been reported to oxidize C1, C4, and C6 positions in cellulose. However, currently no direct evidence exists that PMOs oxidize C6 positions in cellulose, and molecular mechanism of C1, C4 and C6 oxidation is unclear.

**Results:**

In this study, a PMO gene (*Ctpmo1*) belonging to AA9 was isolated from *Chaetomium thermophilum* and successfully expressed and correctly processed in *Pichia pastoris*. A simple and effective chemical method of using Br_2_ to oxidize CtPMO1 reaction products was developed to directly identify C4- and C6-oxidized products by matrix-assisted laser desorption/ionization-time-of-flight tandem mass spectrometry (MALDI-TOF–MS). The PMO (CtPMO1) cleaves phosphoric acid-swollen cellulose (PASC) and celloheptaose, resulting in the formation of oxidized and nonoxidized oligosaccharides. Product identification shows that the enzyme can oxidize C1, C4, and C6 in PASC and cello-oligosaccharides. Mutagenesis of the aromatic residues Tyr27, His64, His157 and residue Tyr206 on the flat surface of CtPMO1 was carried out using site-directed mutagenesis to form the mutated enzymes Y27A, H64A, H157A, and Y206A. It was demonstrated that Y27A retained complete activity of C1, C4, and C6 oxidation on cellulose; Y206A retained partial activity of C1 and C4 oxidation but completely lost activity of C6 oxidation on cellulose; H64A almost completely lost activity of C1, C4, and C6 oxidation on cellulose; and H157A completely lost activity of C1, C4, and C6 oxidation on cellulose.

**Conclusions:**

This finding provides direct and molecular evidence for C1, C4, especially C6 oxidation by lytic polysaccharide monooxygenase. CtPMO1 oxidizes not only C1 and C4 but also C6 positions in cellulose. The aromatic acid residues His64, His157 and residue Tyr206 on CtPMO1 flat surface are involved in activity of C1, C4, C6 oxidation.

**Electronic supplementary material:**

The online version of this article (10.1186/s13068-018-1156-2) contains supplementary material, which is available to authorized users.

## Background

Cellulose is one of the most abundant renewable carbohydrates on earth. Enzymatic degradation of cellulose to glucose has great potential for biofuel production [1]. This enzymatic degradation is thought to be accomplished by the synergistic action of three classes of cellulases: endocellulases, exocellulases, and beta-glucosidases [2]. However, the low degradation efficiency and the high cost of cellulases are major barriers to economical biofuel production [3]. Recently, a new class of cellulose-degrading enzymes, called Cu^2+^-dependent lytic polysaccharide monooxygenases (PMOs) [4], have been discovered and are attracting increasing interest because their oxidative degradation of cellulose dramatically boosts cellulase activity in cellulose hydrolysis [5–8].

Based on their amino acid sequence similarities, PMO enzymes are classified into four families: auxiliary activity 9 (AA9), auxiliary activity 10 (AA10), auxiliary activity 11 (AA11), and auxiliary activity 13 (AA13) [4, 9, 10]. With a plethora of biochemical, structural, functional, and regioselective data available, PMOs from AA9 have been studied intensively in fungi [7, 8, 11–18]. From a structural perspective, the 3-D structure of AA9 PMOs, to perform cleavage, displays a beta-sheet fold with a flat substrate-binding surface that differs from traditional cellulases in that it does not show a substrate-binding cleft (groove, crevice, or tunnel). The flat surface of an AA9 PMO binds cellulose molecules and contains at its center an absolutely conserved N-terminal histidine residue where a copper ion is confirmed to bind tightly [5, 7, 14, 19]. With respect to the oxidation position of substrate molecules, AA9 PMO enzymes have demonstrated striking differences in regioselectivities, most possibly because of their multigenicity [4, 11]. The enzymes oxidize the C1 carbon atom of glucose, but may also oxidize C4 or even C6. Through an elimination reaction, the C1 and C4 oxidation results in two direct cleavage sites of the glucosidic bond. The C1 oxidation leads to the formation of sugar lactone, which is spontaneously hydrolyzed into aldonic acid, and the C4 and C6 oxidations lead to the formation of C4-ketoaldose [8, 11, 12, 16, 17, 20, 21] and C6-hexodialdose [7, 15], respectively. However, no direct evidence demonstrates the presence of C6-hexodialdose in PMO’s reaction products [12, 17]. Although PMO C6 oxidation has been suggested based only on mass measurements [7, 15], mass measurements alone are not enough to confirm that PMOs can oxidize C6 positions of cellulose, because C6-hexodialdose has the same molecular weight as C4-ketoaldose [22]. Because PMO C6 oxidation has not been rigorously demonstrated [12, 17], the described three groups of AA9 PMOs (PMO1 for C1 oxidation, PMO2 for C4 oxidation, and PMO3 for C1 and C4 oxidation) are not involved in C6 oxidation [17]. Currently, PMO C6 oxidation has remained elusive and considerable academic debate is being conducted regarding PMO C6 oxidation [11, 12, 17, 21, 22].

*Chaetomium thermophilum* is a thermophilic fungus and can grow in temperatures up to 60 °C. The thermophilic fungus has been suggested as a model organism for biochemical and prospective structural analyses of eukaryotic macromolecular complexes and biotechnological applications of thermostable eukaryotic proteins [23, 24]. *C*. *thermophilum* genome analysis reveals 19 genes encoding putative AA9 PMOs (http://www.fungalgenomics.cn). In the present paper, we provide direct evidence that an AA9 PMO from *C*. *thermophilum* displays oxidation at C1, C4, and C6 positions of cellulose, especially at C6 positions of cellulose.

## Results

### CtPMO1 expression and purification

We amplified a gene (KC441882) with only one amino acid difference from the Chath2p7_007187 gene of *C*. *thermophilum* genome by polymerase chain reaction (PCR) with a pair of specific primers from the CGMCC3.17990 strain of *C*. *thermophilum* from China (Additional file 1: Table S1). The KC441882 gene was designated as *Ctpmo1*, encoding a putative AA9 PMO protein, CtPMO1. The CtPMO1 protein was predicted to be a secreted enzyme with a 17-amino acid potential signal peptide. The mature CtPMO1 protein is composed of 229 amino acids with a calculated molecular weight of 24.63 kDa. Like most AA9 proteins, CtPMO1 has only a catalytic domain and no additional modules [20].

To obtain the CtPMO1 protein with a native N-terminus, we used the plasmid pPICZαA for *Ctpmo1* expression in *P*. *pastoris*. After induction with methanol, we purified the expressed Cu^2+^-loaded CtPMO1 using nickel affinity chromatography from the Cu^2+^-containing culture filtrate of *P*. *pastoris* transformed with the recombinant plasmid pPICZαA/*Ctpmo1* (Additional file 1: Figure S1). Using SDS-PAGE, we estimated the molecular weight of the purified recombinant CtPMO1, which contains a 6×His tag and a myc tag (2.68 kDa) at the C-terminal, to be approximately 27.5 kDa. Subtracting 2.68 kDa brought this value very close to the 24.63 kDa, we calculated from the deduced amino acid sequence of CtPMO1. We identified the N-terminal amino acid sequence of CtPMO1 using LC-MS/MS to be HAIFQK (Additional file 1: Figure S2), indicating that CtPMO1 was correctly processed in *P*. *pastoris*. As in the case of other PMOs expressed in *P*. *pastoris* [16, 25], the N-terminal His residue in CtPMO1 is not methylated.

### Identification of CtPMO1 soluble reaction products

**Fig. 1:**
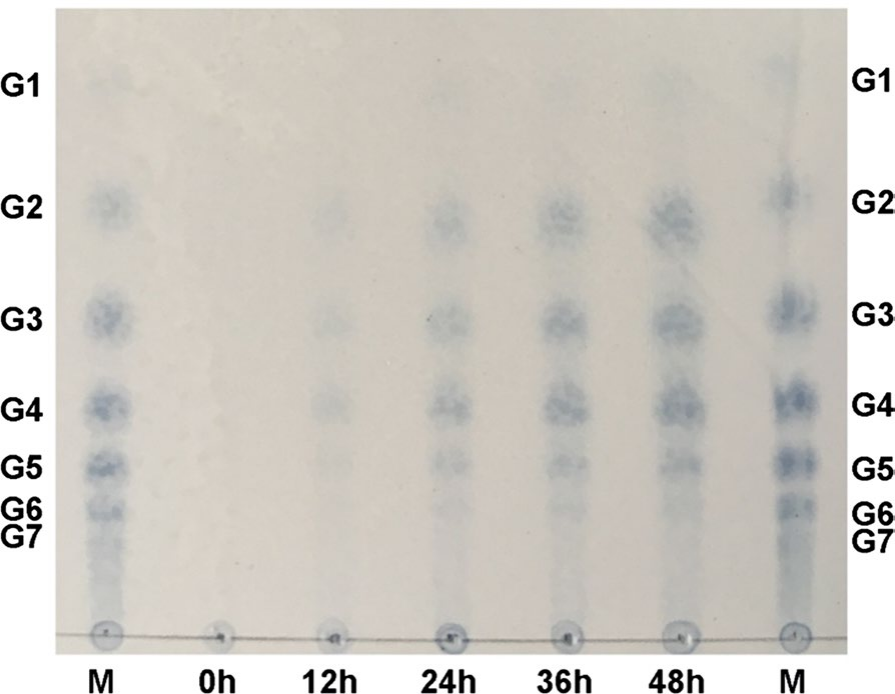
Analysis of CtPMO1 soluble reaction products with PASC as substrate using TLC. Soluble reaction products upon incubation of 0.5% PASC with CtPMO1 in 10 mM HAc-NH_4_Ac (pH 5.0) and 1 mM ascorbate at 50 °C for 0, 12, 24, 36, and 48 h. M, standard cellulo-oligosaccharides (G1–G7)

**Fig. 2:**
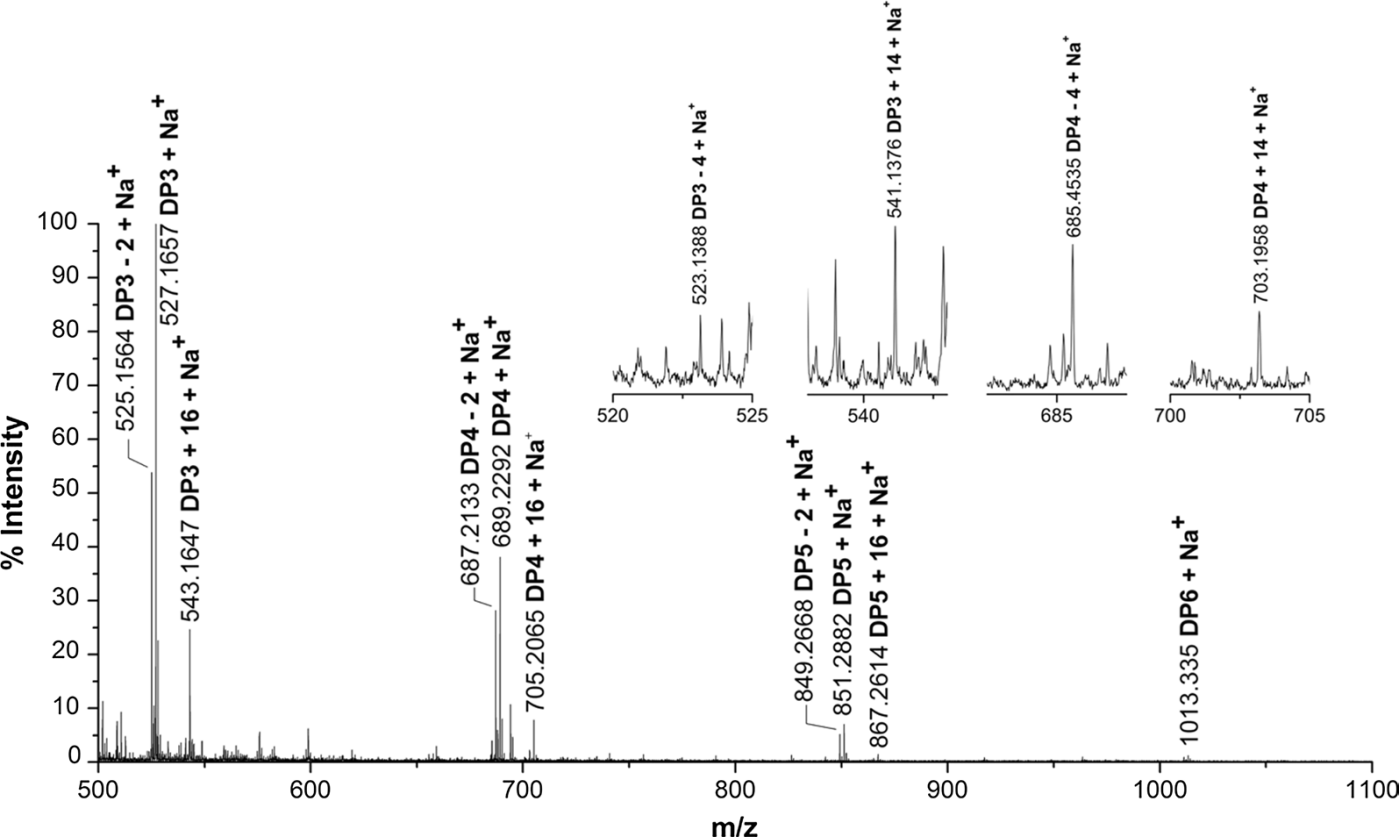
Identification of CtPMO1 soluble reaction products with PASC as substrate using MALDI-TOF–MS. Soluble reaction products upon incubation of 0.5% PASC with CtPMO1 in 10 mM HAc-NH_4_Ac (pH 5.0) and 1 mM ascorbate at 50 °C for 48 h

To identify products of CtPMO1, we performed soluble product assay on phosphoric acid-swollen cellulose (PASC) using TLC and MALDI-TOF–MS analysis. TLC analysis showed that treatment of PASC with CtPMO1 mainly produced cello-oligosaccharides with a degree of polymerization (DP) from DP_2_ to DP_6_ (Fig. 1). MALDI-TOF–MS analysis showed that treatment of PASC with CtPMO1 produced a series of molecular ions with *m/z* corresponding to cello-oligosaccharide products with a DP from DP_3_ to DP_6_ (Fig. 2). Importantly, the molecular ions corresponding to various oxidized oligosaccharides, C1-oxidized oligosaccharides (aldonic acid, *m/z* + 16) and C4- or C6-oxidized oligosaccharides (C4-ketoaldose or C6-hexodialdose, *m/z* − 2), were observed, indicating the nature of oxidative enzymes. Additionally, minor double C4 and C6 oxidized oligosaccharides (*m/z* − 4) and double C1 and C4 or C6 oxidized oligosaccharides (*m/z* + 14) were observed. MS/MS fragmentation of the highest peak with *m/z* value of 525 from MALDI-TOF–MS analysis, corresponding to the C6 or C4-oxidized products (DP_3_-2), showed the presence of nonoxidized fragmentation ions and C6 or C4-oxidized fragmentation ions (Additional file 1: Figure S3, S4, Table S2). In particular, we observed that the potential fragmentation ions oxidized only at C6, indicating that the C6 positions could be modified.

Because NMR has been applied to identify galactose oxidation at C6 positions [26], it was performed to confirm C6-oxidized cellulo-oligosaccharides (C6-hexodialdose) in CtPMO1 soluble reaction products. As expected, ^1^H NMR spectrum of CtPMO1 soluble reaction products displayed an aldehyde proton signal at δ 8.39 (Additional file 1: Figure S5). The anomeric resonance at δ 8.39 is close to the anomeric resonances at δ 9.19 and 9.50 that are assigned to the aldehyde proton of two C6-oxidized galactose products [26].

**Fig. 3:**
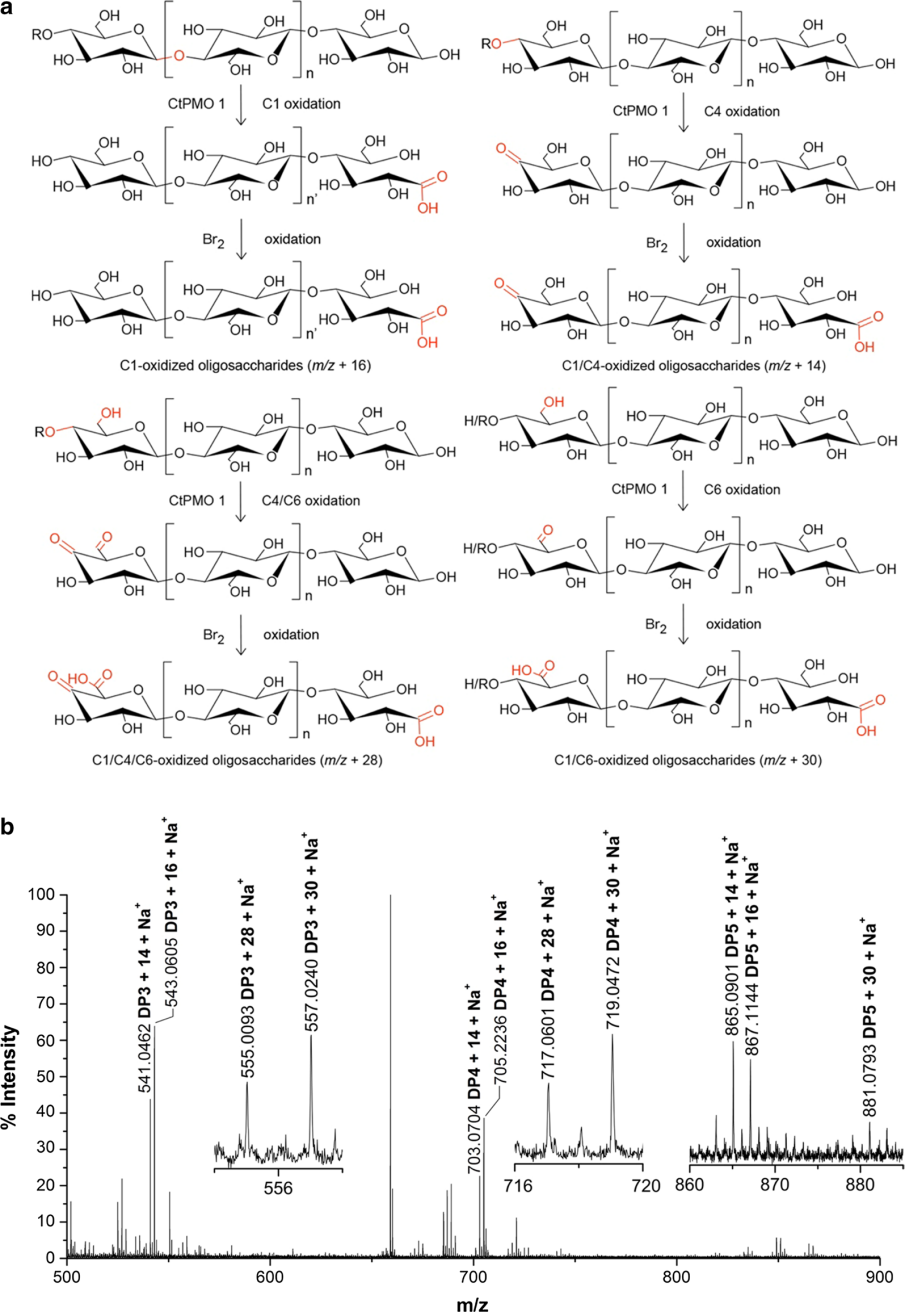
Identification of CtPMO1 soluble reaction products oxidized by Br_2_ with PASC as substrate using MALDI-TOF–MS. Reaction pathway for oxidation of cellulose by CtPMO1 followed Br_2_ oxidation (**a**) and reaction products oxidized by Br_2_ (**b**). C1-oxidized oligosaccharides (*m/z* + 16), C4- and C1-oxidized oligosaccharides (*m/z* + 14), C1- and C6-oxidized oligosaccharides (*m/z* + 30), and C1-, C6- and C4-oxidized oligosaccharides (*m/z* + 28)

To confirm CtPMO1 C4 and C6 oxidation, we performed another chemical method, using Br_2_ to oxidize CtPMO1 soluble reaction products for MALDI-TOF–MS analysis. Based on the chemical method, if CtPMO1 products had C1-oxidized oligosaccharides (*m/z* + 16), they would not be oxidized by Br_2_; if CtPMO1 products had C4-oxidized oligosaccharides (C4-ketoaldose, *m/z* − 2), they would be oxidized by Br_2_ to form C4- and C1-oxidized oligosaccharides (4-keto-aldonic acid, *m/z* + 14); if CtPMO1 products had C6-oxidized oligosaccharides (C6-hexodialdose, *m/z* − 2), they would be oxidized by Br_2_ to form C6- and C1-oxidized cello-oligosaccharides (*m/z* + 30); and if CtPMO1 products had C4- and C6-oxidized oligosaccharides (*m/z* − 4), they would be oxidized by Br_2_ to form C4-, C6- and C1-oxidized cello-oligosaccharides (*m/z* + 28) (Fig. 3a). As expected, we observed the molecular ions corresponding to various oxidized oligosaccharides, C4- and C1-oxidized oligosaccharides (*m/z* + 14), C6- and C1-oxidized oligosaccharides (*m/z* + 30), and C6-, C4-, and C1-oxidized oligosaccharides (*m/z* + 28) in CtPMO1 soluble reaction products oxidized by Br_2_ using MALDI-TOF–MS analysis (Fig. 3b). The proportion of C6-oxidized oligosaccharides (*m/z* + 30) is about 1/10–1/5 of C4-oxidized oligosaccharides (*m/z* + 14), and the proportion of C6-oxidized oligosaccharides (*m/z* + 30) and C4- and C6-oxidized oligosaccharides (*m/z* + 28) is about 1/6–1/3 of C4-oxidized oligosaccharides (*m/z* + 14), indicating that C4-oxidized oligosaccharides is predominant in CtPMO1 soluble reaction products.

### CtPMO1 C4- and C1-oxidized products are also present in its insoluble reaction products

**Fig. 4:**
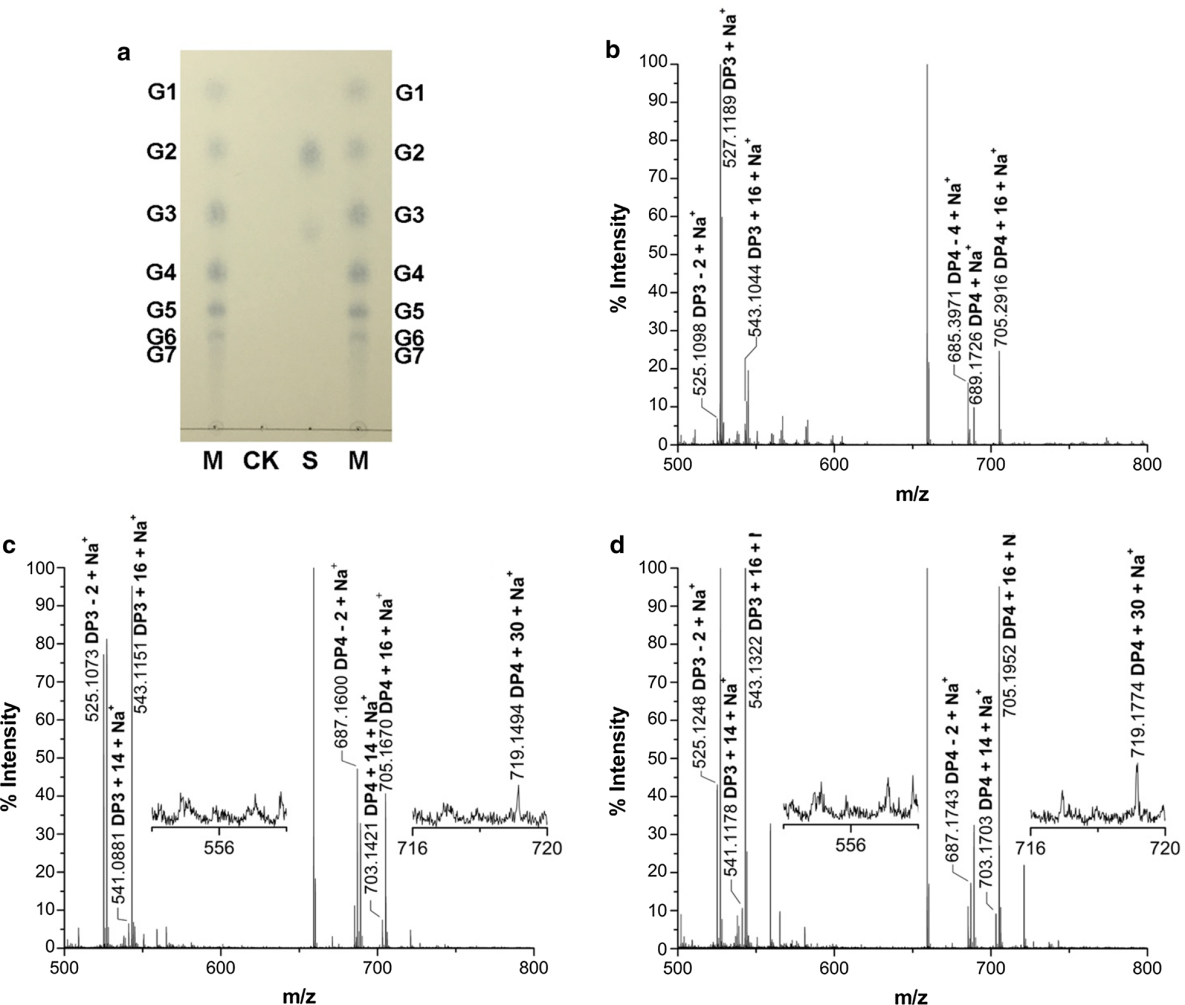
CtPMO1 insoluble reaction products hydrolyzed by endo-1,4-beta-glucanase followed by Br_2_ oxidation with PASC as substrate. Insoluble reaction products (residual PASC) upon incubation of 0.5% PASC with CtPMO1 in 10 mM HAc-NH_4_Ac (pH 5.0) and 1 mM ascorbate at 50 °C for 48 h. CtPMO1 insoluble reaction products hydrolyzed by endo-1,4-beta-glucanase using TLC analysis (**a**) and MALDI-TOF–MS (**b**). CtPMO1 insoluble reaction products hydrolyzed by endo-1,4-beta-glucanase followed by Br_2_ oxidation for 30 min (**c**) and for 60 min (**d**) using MALDI-TOF–MS analysis. C1-oxidized oligosaccharides (*m/z* + 16), C4- or C6-oxidized oligosaccharides (*m/z* − 2), lactones (*m/z* -4) of C1- (*m/z* + 16) and C4- or C6-oxidized oligosaccharides (*m/z* − 2), C4- and C1-oxidized oligosaccharides (*m/z* + 14), C6- and C1-oxidized oligosaccharides (*m/z* + 30), and C6-, C1- and C4-oxidized oligosaccharides (*m/z* + 28). M, standard cellulo-oligosaccharides (G1–G7). CK, samples upon incubation of insoluble reaction products (residual PASC) in 10 mM HAc-NH_4_Ac (pH 5.0) at 50 °C for 10 min with inactive endo-1,4-beta-glucanase treated at 100 °C for 30 min

To confirm whether CtPMO1 products also exist in its insoluble reaction products (residual PASC), CtPMO1 insoluble reaction products hydrolyzed by endo-1,4-beta-glucanase followed by Br_2_ oxidation were identified using MALDI-TOF–MS analysis (Fig. 4). Just as expected, nonoxidized cello-oligosaccharides (*m/z* + 0), C1-oxidized oligosaccharides (*m/z* + 16) and C4- or C6-oxidized oligosaccharides (*m/z* − 2) were released from CtPMO1 insoluble reaction products hydrolyzed by endo-1,4-beta-glucanase (Fig. 4a, b). Unexpectedly, oxidized oligosaccharides with DP_4_ (*m/z* − 4) were obviously observed in CtPMO1 insoluble reaction products hydrolyzed by endo-1,4-beta-glucanase. After Br_2_ oxidation, we observed the main molecular ions corresponding to C1-oxidized oligosaccharides (*m/z* + 16) and C4- and C1-oxidized oligosaccharides (*m/z* + 14) and the minor molecular ions corresponding to C6- and C1-oxidized oligosaccharides with DP_4_ (*m/z* + 30), but failed to observe molecular ions corresponding to C6- and C1-oxidized oligosaccharides with DP_3_ (*m/z* + 30) and C6-, C4-, and C1-oxidized oligosaccharides (*m/z* + 28), in CtPMO1 insoluble reaction products hydrolyzed by endo-1,4-beta-glucanase followed by Br_2_ oxidation for 30 min (Fig. 4c) and 60 min (Fig. 4d). The absence of the molecular ions corresponding to C6-, C4-, and C1-oxidized oligosaccharides (*m/z* + 28) shows that the oxidized oligosaccharides with DP_4_ (*m/z* − 4) in CtPMO1 insoluble reaction products hydrolyzed by endo-1,4-beta-glucanase are lactones (*m/z* − 2) of C4 or C6-oxidized (*m/z* − 2) aldonic acid (*m/z* + 16) rather than C6- and C4-oxidized oligosaccharides (*m/z* − 4). These data indicate that C6-oxidized products are minor in CtPMO1 insoluble reaction products. It should be pointed out that the molecular ion peaks (*m/z* − 2) dramatically increased, but the molecular ion peaks (*m/z* + 14 and + 30) did not obviously increase, in CtPMO1 insoluble reaction products hydrolyzed by endo-1,4-beta-glucanase followed by Br_2_ oxidation. The most possible reason for this is that the molecular ion peaks (*m/z* − 2) correspond to lactones (*m/z* − 2) of C1-oxidized oligosaccharides (aldonic acid, *m/z* + 16), which are largely produced from nonoxidized oligosaccharides by Br_2_ oxidation and easily converted to their lactones (*m/z* − 2) in solution.

### CtPMO1 can C1-, C4-, and C6-oxidize soluble celloheptaose

**Fig. 5:**
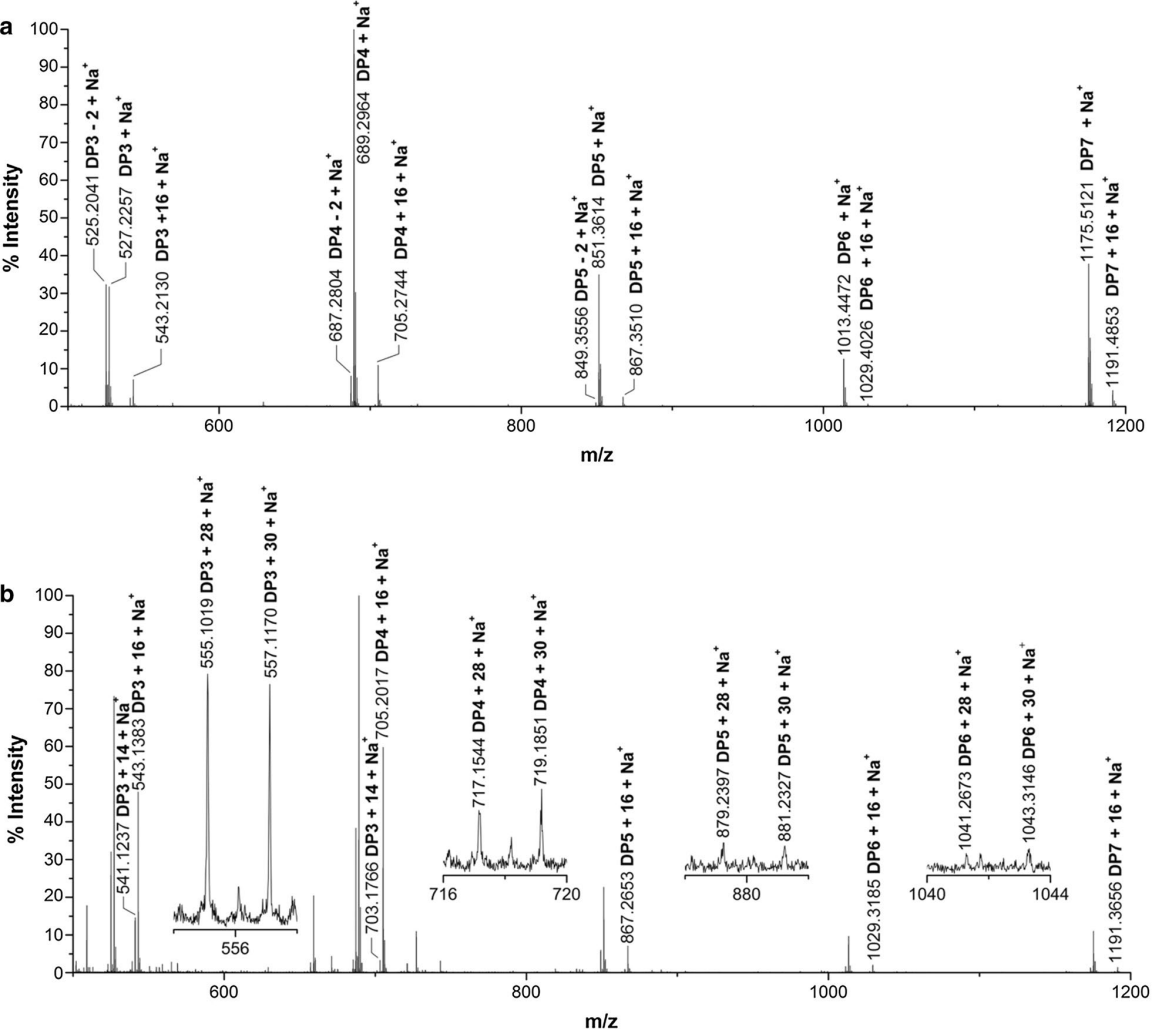
Identification of CtPMO1 reaction products oxidized by Br_2_ with celloheptaose as substrate using MALDI-TOF–MS. Reaction products upon incubation of 0.1% celloheptaose with CtPMO1 in 10 mM HAc-NH_4_Ac (pH 5.0) and 1 mM ascorbate at 50 °C for 48 h. Reaction products (**a**) and reaction products oxidized by Br_2_ (**b**). C1-oxidized oligosaccharides (*m/z* + 16), C4- or C6-oxidized oligosaccharides (*m/z* − 2), C4- and C1-oxidized oligosaccharides (*m/z* + 14), C6- and C1-oxidized oligosaccharides (*m/z* + 30), and C1-, C6- and C4-oxidized oligosaccharides (*m/z* + 28)

To confirm C1, C4, and C6 oxidation of CtPMO1 on soluble cello-oligosaccharides, we used celloheptaose as substrates of CtPMO1 to generate oxidized cellulo-oligosaccharides. MALDI-TOF–MS/MS analysis confirmed the presence of C1-oxidized oligosaccharides (aldonic acid, *m/z* + 16) and C6- or C4-oxidized oligosaccharides (C6-hexodialdose or C4-ketoaldose, *m/z* − 2) upon incubating CtPMO1 with celloheptaose (Fig. 5a). Like CtPMO1 soluble reaction products oxidized by Br_2_ with PASC as substrate, we also observed the molecular ions corresponding to C4- and C1-oxidized oligosaccharides (*m/z* + 14), C6- and C1-oxidized oligosaccharides (*m/z* + 30), and C6-, C4- and C1-oxidized oligosaccharides (*m/z* + 28), in CtPMO1 reaction products oxidized by Br_2_ with celloheptaose as substrate using MALDI-TOF–MS analysis (Fig. 5b), indicating that CtPMO1 can oxidize C4, C1 and C6 positions of soluble oligosaccharides.

### Structural model of CtPMO1

To understand the molecular basis of CtPMO1 C1, C4 and C6 oxidation, the 3-D structure of its catalytic domain was predicted on the basis of known 3-D structures of PMO proteins using homology modeling [5, 7, 14, 19, 27]. CtPMO1 shares a high identity of 64.32% with a PMO (NcLPMO9C) of *Neurospora crassa* (Additional file 1: Figure S6), whose 3-D structure (PDB id 4D7U) has been reported [27]. Therefore, the homology model of CtPMO1 was obtained using NcLPMO9C as a template. The homology model shows that three highly conserved amino acid residues, His1, His83 and Tyr168, are clustered at the flat surface near the N-terminus of CtPMO1, and four aromatic residues Tyr27, His64, His157 and Tyr206 (two conserved residues His157 and Tyr206, and two varied residues Tyr27, His64) are present on the flat surface of CtPMO1 (Additional file 1: Figure S7). The N-terminal amino group (2.3 Å), the Nδ of His1 (2.2 Å), and the Nε of His83 (2.0 Å) form a copper ion-binding site (a histidine-brace). The highly conserved residue Tyr168 is buried and lies in the protein-facing axial position with a distance of 3.0 Å from the copper to the oxygen atom of the Tyr168 side chain. The three highly conserved residues His1, His83, and Tyr168 coordinate the copper ion essential for catalysis [11, 27]. The role in catalytic activity of the aromatic residues Tyr27, His64, His157, and Tyr206 on the flat surface of CtPMO1 remains unclear (Additional file 1: Figure S8).

### Role of the aromatic residues Tyr27, His64, His157 and Tyr206 on the flat surface of CtPMO1

**Fig. 6:**
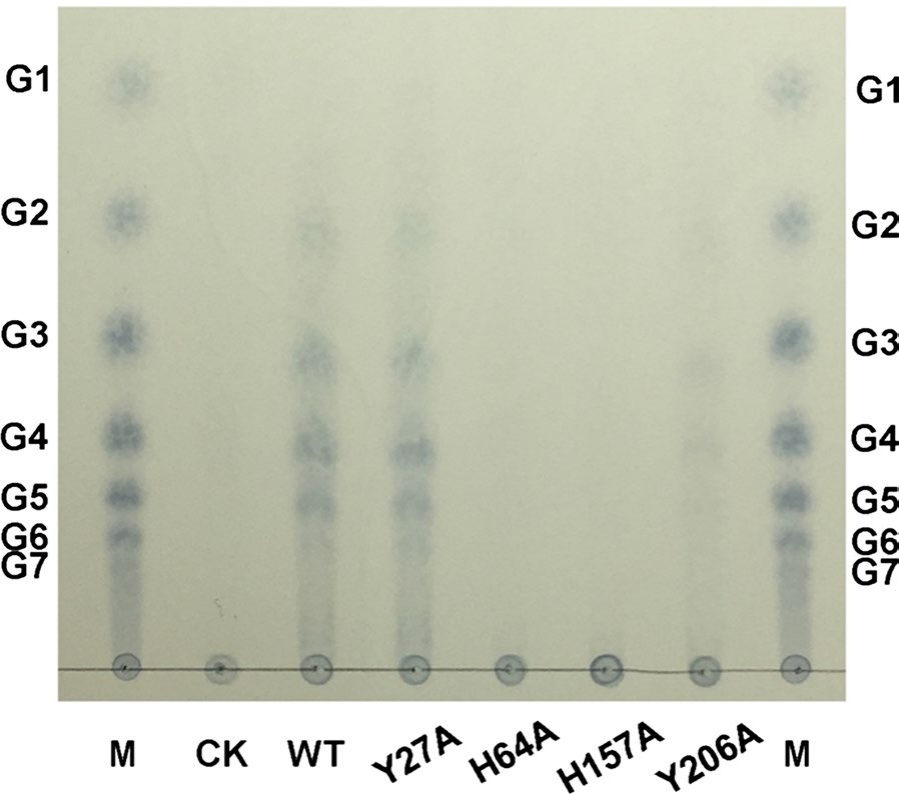
Identification of the mutated CtPMO1 soluble reaction products with PASC as substrate using TLC. Soluble reaction products upon incubation of 0.5% PASC with the mutated CtPMO1 enzymes (Y27A, H64A, H157A and Y206A) in 10 mM HAc-NH_4_Ac (pH 5.0) and 1 mM ascorbate at 50 °C for 48 h. M, standard cellulo-oligosaccharides (G1–G7). CK, samples upon incubation of 0.5% PASC in 10 mM HAc-NH_4_Ac (pH 5.0) and 1 mM ascorbate at 50 °C for 48 h with inactive CtPMO1 treated at 100 °C for 30 min

**Fig. 7:**
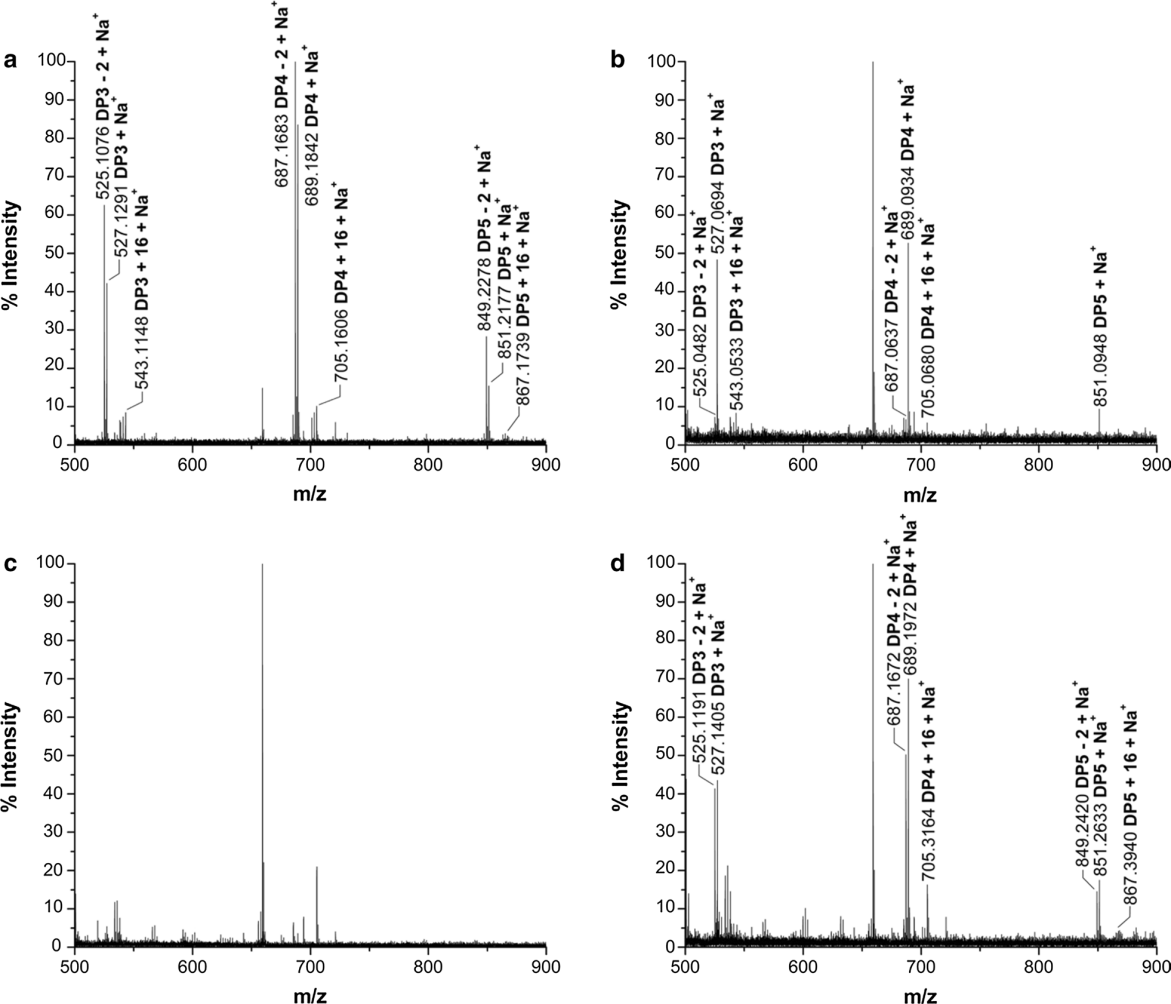
Identification of the mutated CtPMO1 soluble reaction products with PASC as substrate using MALDI-TOF–MS. Soluble reaction products upon incubation of 0.5% PASC with the mutated CtPMO1 enzymes Y27A (**a**), H64A (**b**), H157A (**c**) and Y206A (**d**) in 10 mM HAc-NH_4_Ac (pH 5.0) and 1 mM ascorbate at 50 °C for 48 h. C1-oxidized oligosaccharides (aldonic acid, *m/z* + 16) and C4- or C6-oxidized oligosaccharides (C4-ketoaldose or C6-hexodialdose, *m/z* − 2)

To determine whether the aromatic residues Tyr27, His64, His157 and Tyr206 on the flat surface of CtPMO1 are involved in catalytic activity of C1, C4 and C6 oxidation, mutation of the four residues of the wild-type CtPMO1 enzyme (WT) was carried out using site-directed mutagenesis to form four mutated enzymes: Y27A, H64A, H157A and Y206A. TLC analysis of soluble reaction products showed that cello-oligosaccharides were observed in Y27A, a minor amount of cello-oligosaccharides were observed in Y206A, and no cello-oligosaccharides were observed in H64A and H157A (Fig. 6). MALDI-TOF–MS analysis showed that C1-oxidized (*m/z* + 16) and C4- or C6-oxidized (*m/z* − 2) cello-oligosaccharides were observed in Y27A and Y206A, a minor amount of C1-oxidized (*m/z* + 16) and C4- or C6-oxidized (*m/z* − 2) cello-oligosaccharides were observed in H64A, and no oxidized cello-oligosaccharides were observed in H157A (Fig. 7).

**Fig. 8:**
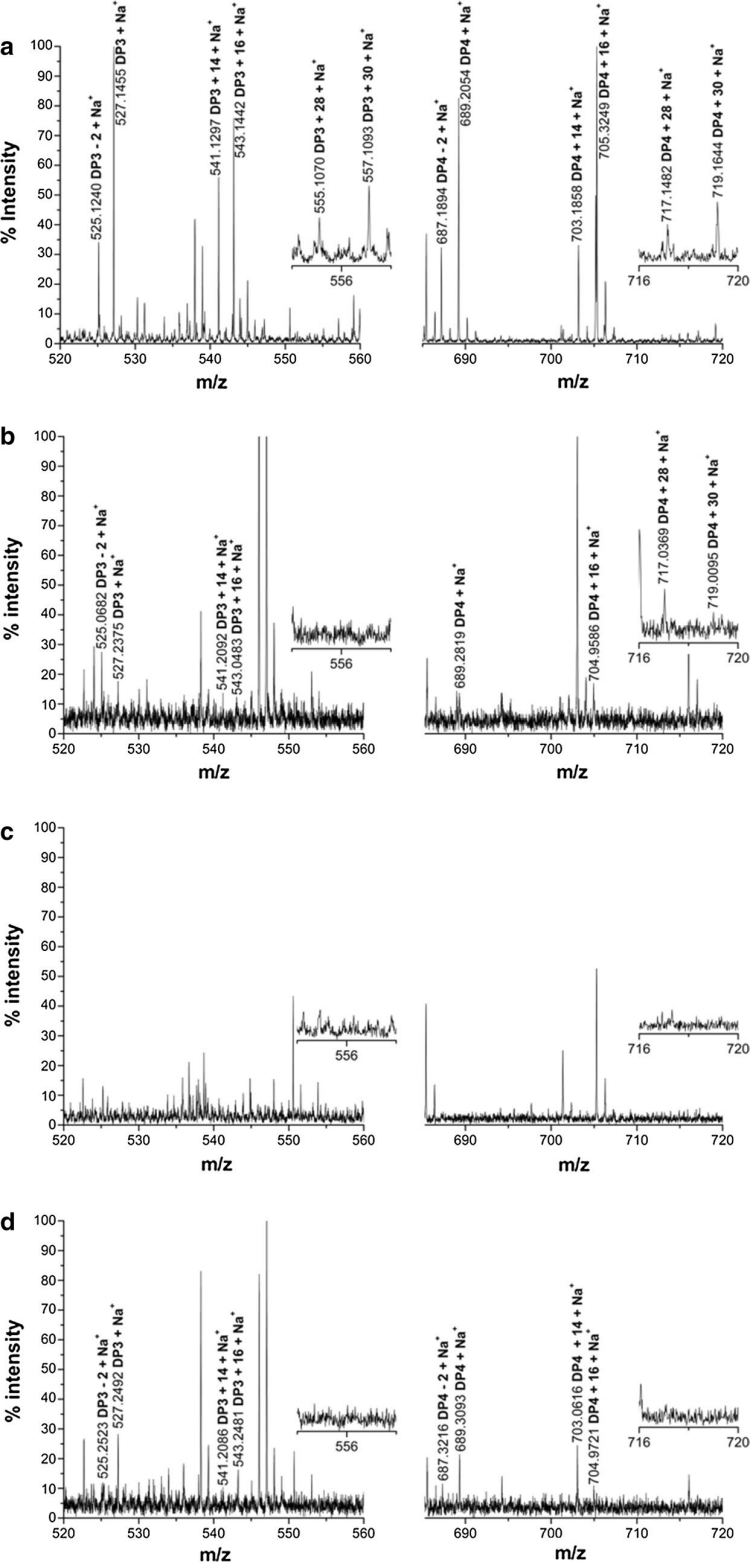
Identification of the mutated CtPMO1 soluble reaction products oxidized by Br_2_ with PASC as substrate using MALDI-TOF–MS. Soluble reaction products upon incubation of 0.5% PASC with the mutated CtPMO1 enzymes Y27A (**a**), H64A (**b**), H157A (**c**) and Y206A (**d**) in 10 mM HAc-NH_4_Ac (pH 5.0) and 1 mM ascorbate at 50 °C for 48 h. C1-oxidized oligosaccharides (*m/z* + 16), C4-oxidized oligosaccharides (*m/z* + 14), C6-oxidized oligosaccharides (*m/z* + 30), and C6- and C4-oxidized oligosaccharides (*m/z* + 28)

**Fig. 9:**
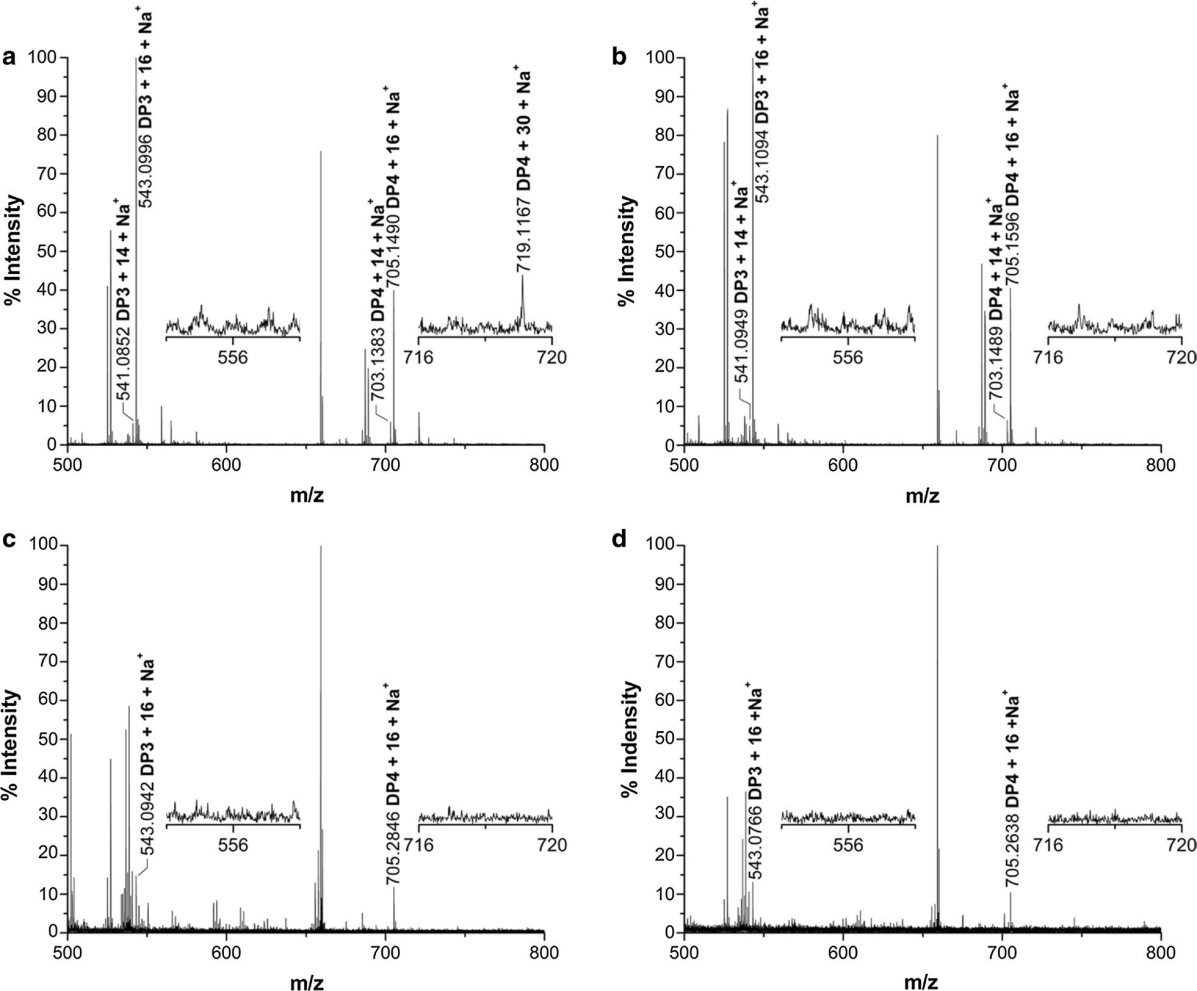
Mutated CtPMO1 insoluble reaction products oxidized by Br_2_ with PASC as substrate using MALDI-TOF–MS. Insoluble reaction products (residual PASC) upon incubation of 0.5% PASC with mutated CtPMO1 Y27A (**a**), H64A (**b**), H157A (**c**) and Y206A (**d**) in 10 mM HAc-NH_4_Ac (pH 5.0) and 1 mM ascorbate at 50 °C for 48 h. C1-oxidized oligosaccharides (*m/z* + 16), C4-oxidized oligosaccharides (*m/z* + 14), and C6-oxidized oligosaccharides (*m/z* + 30)

To confirm the involvement of the residues Tyr27, His64, His157 and Tyr206 of CtPMO1 in C4 and C6 oxidation, soluble reaction products of the mutated enzymes were oxidized by Br_2_ for MALDI-TOF–MS analysis as the WT enzyme. As expected, C4- and C1-oxidized oligosaccharides (*m/z* + 14), C6- and C1-oxidized oligosaccharides (*m/z* + 30), and C1-, C4- and C6-oxidized oligosaccharides (*m/z* + 28) were observed in Y27A, a minor amount of C4- and C1-oxidized oligosaccharides (*m/z* + 14), C6- and C1-oxidized oligosaccharides (*m/z* + 30), and C1-, C4- and C6-oxidized oligosaccharides (*m/z* + 28) were observed in H64A, a minor amount of C4- and C1-oxidized oligosaccharides (*m/z* + 14) but no C6- and C1-oxidized oligosaccharides (*m/z* + 30) and C1-, C4- and C6-oxidized oligosaccharides (*m/z* + 28) were observed in Y206A, and no oxidized oligosaccharides were observed in H157A (Fig. 8). Similar analysis of insoluble reaction products showed that C4- and C1-oxidized oligosaccharides (*m/z* + 14) and C6- and C1-oxidized oligosaccharides (*m/z* + 30) were observed in Y27A, a minor amount of C4- and C1-oxidized oligosaccharides (*m/z* + 14) were observed in H64A, and no C4- and C1-oxidized oligosaccharides (*m/z* + 14) and C6- and C1-oxidized oligosaccharides (*m/z* + 30) were observed in H157A and Y206A (Fig. 9).

Together, these data indicate that the mutated enzyme Y27A retained complete activity of C1, C4, and C6 oxidation on cellulose; the mutated enzyme Y206A retained partial activity of C1 and C4 oxidation but completely lost activity of C6 oxidation on cellulose; the mutated enzyme H64A almost completely lost activity of C1, C4, and C6 oxidation on cellulose; and the mutated enzyme H157A completely lost activity of C1, C4, and C6 oxidation on cellulose (Table 1), suggesting that the residue His157 in CtPMO1 is required for activity of C1, C4, and C6 oxidation on cellulose; the residue His64 in CtPMO1 plays a key role in retaining activity of C1, C4, and C6 oxidation on cellulose; the residue Tyr206 in CtPMO1 plays a partial role in retaining activity of C1 and C4 oxidation but a key role in retaining activity of C6 oxidation on cellulose; and the residue Tyr27 in CtPMO1 may not play an important role in retaining activity of C1, C4, and C6 oxidation on cellulose.

**Table 1:** Composition of CtPMO1 and its mutants’ reaction products with PASC as substrate

Enzyme	Soluble reaction products				Insoluble reaction products			
	C1	C4	C6	C4 + C6	C1	C4	C6	C4 + C6
WT	+++	+++	+++	+++	+++	+++	+	−
Y27A	+++	+++	+++	+++	+++	+++	+	−
H64A	+	+	+	+	+	+	−	−
H157A	−	−	−	−	−	−	−	−
Y206A	+	+	−	−	+	−	−	−

“+++”: products can be detected; “+”: minor products can be detected; “−”: no products

## Discussion

Cello-oligosaccharides containing a C6 aldehyde can be oxidized by I_2_ and Br_2_ to the corresponding uronic acids [12]. In this study, we developed a simple and effective chemical method, using Br_2_ to oxidize CtPMO1 reaction products for identifying C4- and C6-oxidized products. The method has three potential advantages. First, because there is no interference by metal ions (Na^+^ and Ag^+^), Br_2_ oxidation of CtPMO1 products allows us to directly identify C4- and C6-oxidized products using MALDI-TOF–MS analysis, but there is interference of metal ions (Na^+^ and Ag^+^) in CtPMO1 products oxidized by I_2_. Second, residual Br_2_ in CtPMO1 products oxidized by Br_2_ is very easily removed under a stream of nitrogen at 40 °C, unlike the removal of I_2_ which requires adding excess Ag_2_CO_3_ in CtPMO1 products oxidized by I_2_. Third, Br_2_ oxidation of CtPMO1 reaction products provided acidic conditions that can prevent the generation of unsaturated oligosaccharides, unlike oxidized by I_2_ under alkali conditions.

CtPMO1 oxidizes PASC to produce C1-, C4-, and C6-oxidized products in its soluble reaction products and C4- and C1-oxidized products in its insoluble reaction products, but C6-oxidized products are minor in its insoluble reaction products. One possible explanation for this is that C6-oxidized oligosaccharides in CtPMO1 soluble reaction products may be mainly produced by C6 oxidation of C4- and C1-cleaved soluble oligosaccharides (oxidized and nonoxidized oligosaccharides). This explanation is supported by the evidence that CtPMO1 C6-oxidizes soluble celloheptaose to produce C6-oxidized oligosaccharides and that LsAA9A and CvAA9A C4-oxidize small oligosaccharides, whereas they C4- and C1-oxidize polysaccharides [28]. Conformational flexibility of soluble cello-oligosaccharides possibly causes the changes in oxidation type (C1, C4, and C6) of a PMO enzyme. It should be pointed out that three PMOs (TaGH61A, PaGH61B and CtPMO1) identified to oxidize C6 position of cellulose ([7, 15], this study) have a long reaction time (TaGH61A for 22 h, PaGH61B for 48 h and CtPMO1 for 48 h) to produce C6-oxidized oligosaccharides. The long reaction time also hints that C6-oxidized oligosaccharides may be produced from C4- and/or C1-oxidize polysaccharides by PMO C6 oxidation.

It has been suggested that aromatic residues on the PMO protein flat surface are involved in substrate binding [14, 27, 28]. In this study, mutation of the residue His157 in CtPMO1 results in complete loss of activity of C1, C4, and C6 oxidation on cellulose, maybe because the residue His157 is adjacent to the copper ion-binding site of CtPMO1. Interestingly, mutation of the residue His64 in CtPMO1 results in almost complete loss of activity of C1, C4, and C6 oxidation on cellulose. The residue His64 lies in a sequence insertion (L3 loop) that seems unique for C4-oxidizing PMOs [27]. It has been reported that an additional metal-binding site (copper or zinc ion) is coordinated by the residue His64 in NcLPMO9C from *N*. *crass* [27]. Recent studies of LsAA9A-substrate interaction show that a hydrogen bond is present between the residue His66 in LsAA9A (His64 in CtPMO1 and NcLPMO9C) and O3 at substrate + 2 [28]. These data suggest that the residue His (His64 in CtPMO1 and NcLPMO9C, His66 in LsAA9A) plays a role in PMO–substrate interaction. Mutation of the residue Tyr206 in CtPMO1 results in partial loss of activity of C1 and C4 oxidation and complete loss of C6 oxidation on cellulose, may be because the residue Tyr206 is far away from the copper ion-binding site (at subsite − 3), suggesting that it may function as a carbohydrate-binding module to enhance binding affinity. The evidence that stacking of Tyr203 in LsAA9A (Tyr206 in CtPMO1) is a major interaction in LsAA9A:Cell5 supports this suggestion [28]. Mutation of the residue Tyr27 in CtPMO1 results in no loss of activity of C1, C4, and C6 oxidation on cellulose, perhaps because the residue Tyr27 is far away from the copper ion-binding site. Because of the absence of structural data of the residue Tyr27, whether it plays a role in CtPMO1-substrate interaction needs be further studied in the future.

It has been suggested that there are different modes for substrate binding by PMOs, inter-chain binding modes and intra-chain binding modes, which allow the active site of PMOs to be close to hydrogen of C1, C4, and C6 carbon of cellulose [14]. Recent studies of PMO–substrate interaction show that the active site of LsAA9A from *Lentinus similis* is close to hydrogen of C1, C4, and C6 carbon of the soluble Cell5 substrate [28]. These structural data support C1, C4, and C6 oxidation of PMOs on cellulose and cello-oligosaccharides.

It is unclear how CtPMO1 oxidizes C6 carbon of cello-oligosaccharides. To our knowledge, only galactose oxidase (EC 1.1.3.9) is a single C6-oxidizing copper metalloenzyme that catalyses C6 oxidation of galactose and its derivatives [4, 26, 29]. Although the overall sequence similarity is low between galactose oxidase and PMO, the two enzymes have very similar active sites. Four highly conserved amino acid side chains (from Tyr272, Tyr495, His496, and His581) directly coordinate copper in a galactose oxidase and the residue Tyr495 is buried and lies in the protein-facing axial position with a distance of 2.6 Å from the copper to the oxygen atom of the Tyr459 side chain [29], similar to the highly conserved and buried residue Tyr168 within CtPMO1.

C6 oxidation of PMOs is interesting because, unlike C1 and C4 oxidation, it cannot directly cleave the glycosidic bond of cellulose. CtPMO1 C6-oxidizes soluble oligosaccharides to produce C6-oxidized oligosaccharides (C6-hexodialdose). It is possible that an unknown mechanism (e.g., a secreted oxidoreductase) might further oxidize C6-hexodialdose generated by PMOs to form glucuronic acid-containing cello-oligosaccharides as I_2_ oxidizes C6-hexodialdose. We hypothesize that there may be two possible enzymatic reactions to degrade glucuronic acid-containing cello-oligosaccharides. One is beta-elimination by polysaccharide lyase. It is well known that polysaccharide lyase family 20 endo-beta-1,4-glucuronan lyases can cleave the glycosidic bond of a glucuronic acid-containing cello-oligosaccharide via beta-elimination [30–32]. The other is hydrolysed by β-glucuronidase and β-glucosidase. The two enzymes can alternately hydrolyze glucuronic acid-containing cello-oligosaccharides to yield glucuronic acid [33, 34]. As an important organic acid, glucuronic acid may act as a chelate required for manganese peroxidase to stimulate its activity by stabilizing Mn^3+^ for depolymerization of lignin [35], or it may be metabolized by the uronic acid pathway in cells to produce important active substances, such as ascorbate (Vc) and d-xylulose [32, 36].

Fungal oxidative degradation of cellulose is complex in nature. Recent genomic sequencing shows multigenicity of AA9 genes in fungi [4]. One of the most extreme examples is that *Coprinopsis cinerea* contains 33 putative AA9 genes [37]. Two thermophilic fungi *Thielavia terrestris* and *Myceliophthora thermophila* contain 24 and 20 putative AA9 genes, respectively [38]. The thermophilic fungus *C. thermophilum* contains 19 putative AA9 genes (http://www.fungalgenomics.cn). These data suggest that AA9 proteins have diverse functions, including regioselectivity diversity.

## Conclusions

CtPMO1 was successfully expressed and correctly processed in *P. pastoris*. A simple and effective chemical method to directly identify C4- and C6-oxidized products by Br_2_ oxidation. CtPMO1 can cleave PASC and celloheptaose, and product identification shows that it also can oxidize three carbon positions in PASC and cello-oligosaccharides so belonging to a C1-, C4- and C6-oxidizing PMO. The mutants of CtPMO1 demonstrated that Y27A retained complete activity of C1, C4, C6 oxidation, indicating Tyr27 effects little to activity of C1, C4, C6 oxidation; Y206A retained partial activity of C1 and C4 oxidation but completely lost activity of C6 oxidation, indicating that Tyr206 mainly affects activity of C6 oxidation with partial impact on activity of C1 and C4 oxidation; H64A almost completely lost activity of C1, C4, C6 oxidation, indicating His64’s importance in C1, C4, C6 oxidation; H157A completely lost activity of C1, C4, C6 oxidation, indicating that His157 has a crucial role in the overall activity of CtPMO1.

## Methods

### Strains, plasmids, culture media, and chemicals

*Chaetomium thermophilum* CGMCC3.17990 strain was previously isolated in China and deposited in the publicly accessible culture collection CGMCC (Beijing, China). We purchased the plasmid vector pPICZαA and *Pichia pastoris* GS115 strain from Invitrogen. For total RNA isolation, we grew *C. thermophilum* at 50 °C for 48 h in a medium containing 2% avicel, 0.4% yeast extract, 0.1% K_2_HPO_4_·3H_2_O, and 0.05% MgSO_4_·7H_2_O, dissolved in tap water. Avicel PH-101, glucose, gluconic acid, and ascorbate were from Sigma-Aldrich. Cellodextrin oligosaccharide mixture and cellopentaose were from Elicityl (Crolles, France). Other reagents were of analytic grade.

### Molecular cloning of cDNA

We used Trizol reagent (Gibco) for total RNA isolation of *C. thermophilum* from mycelia. We performed RT-PCR with RNA PCR Kit 3.0 instruction (Takara). We used PCR to amplify the cDNA of the CtPMO1 protein, termed *Ctpmo1*, with a pair of specific oligonucleotide primers (*Ct*PMO1-cF/*Ct*PMO1-cR) synthesized based on the gene (KC441882) from the genomic sequencing of *C*. *thermophilum* (Additional file 1: Table S1).

### Construction of Ctpmo1 expression vector

We used PCR to amplify the *Ctpmo1* fragment of the coding region without a signal peptide sequence with a pair of specific primer (*Ct*PMO1-F/*Ct*PMO1-R), which contained an *Xho*I and an *Xba*I restriction site, respectively (Additional file 1: Table S1). The PCR product was digested with *Xho*I and *Xba*I and ligated with pPICZαA, yielding the expression plasmid pPICZαA/*Ctpmo1*, which ensured the expression of CtPMO1 in *P. pastoris* with a native N-terminus (Invitrogen). Through DNA sequencing, we confirmed that the constructed recombinant plasmid pPICZαA/*Ctpmo1* contained the *Ctpmo1* sequence.

### Transformation of *P. pastoris*

After linearized with *Sac*I, we transformed the recombinant plasmid pPICZαA/*Ctpmo1* to *P. pastoris* GS115 by electroporation with BTX ECM830 Electroporator (Harvard Apparatus). We selected the transformants on YPDS plates containing 100 mg/L zeocin and verified them with PCR amplifications and DNA sequencing (Invitrogen).

### CtPMO1 induction and purification

We induced the CtPMO1 protein in transformed *P*. *pastoris* with the *Pichia* Expression System Kit (Invitrogen). The transformed *P*. *pastoris* was cultured at 28 °C for 6 days in a shake flask in BMMY medium containing 1 mM Cu^2+^. We centrifuged 1000 mL of the culture filtrate at 10,000*g* at 4 °C for 15 min. To the supernatant, we added (NH_4_)_2_SO_4_ to 90% saturation and gently stirred and kept the solution for 12 h at 4 °C. We collected the resulting precipitate by centrifuging it at 10,000*g* at 4 °C for 15 min, then dissolved it in 50 mM phosphate buffered saline buffer (pH 7.4), and dialyzed it overnight at 4 °C against at least three changes of the same buffer. We purified the C-terminal histidine-tagged CtPMO1 protein through affinity chromatography on a His Trap column (GE Healthcare) with the following steps: balanced His Trap column with buffer A (300 mM NaCl, 2.7 mM KCl, 10 mM K_2_HPO_4_, 2 mM KH_2_PO_4_, 10 mM imidazole, pH 7.4), then loaded the crude enzyme followed by rebalancing the column with buffer B (300 mM NaCl, 2.7 mM KCl, 10 mM K_2_HPO_4_, 2 mM KH_2_PO_4_, 30 mM imidazole, pH 7.4), and eluted CtPMO1 protein by buffer C (300 mM NaCl, 2.7 mM KCl, 10 mM K_2_HPO_4_, 2 mM KH_2_PO_4_, 250 mM imidazole, pH 7.4). The purified protein was pooled and dialyzed fractions overnight at 4 °C against three changes of 10 mM HAc-NH_4_Ac buffer (pH 5.0). We used the purified and desalted Cu^2+^-loaded CtPMO1 protein for further functional studies.

### Protein determination and SDS-PAGE

We used the Lowry method for protein determination [39], determining the purity of the CtPMO1 protein using SDS-PAGE [40].

### The N-terminal amino acid sequence analysis of CtPMO1 protein

We applied LC–MS/MS to determine the N-terminal amino acid sequence of CtPMO1. We performed in-gel tryptic digestion of the purified CtPMO1 with the method previously described [41], extracting and analyzing the resulting peptides with a nano-LC combined with Q Exactive mass spectrometer (Thermo Scientific) in the positive ion mode [42]. We acquired MS and MS/MS spectra on the mass range of m/z range of 300–1800 and 100–1000, respectively. We analyzed all data using MASCOT 2.2 software (Matrix Science) and searched MS/MS spectra against the CtPMO1 protein sequence database.

### Activity assay

We used PASC as substrate, prepared from Avicel according to the method previously described [7]. Assays contained 5 mg/mL (0.5%) PASC or 1 mg/mL (0.1%) cellopentaose and 5 μM Cu^2+^-loaded CtPMO1 protein in 10 mM HAc-NH_4_Ac (pH 5.0) and 1 mM ascorbate for 48 h at 50 °C. When PASC used as substrate, the reaction mixture was centrifuged at 10,000*g* at 4 °C for 10 min. The supernatant was recovered for analysis of soluble reaction products of CtPMO1. The precipitate (residual PASC) was washed with water three times and was finally suspended in 10 mM HAc-NH_4_Ac (pH 5.0). To release oxidized oligosaccharides from insoluble reaction products of CtPMO1, the residual PASC was hydrolyzed with endo-1,4-beta-glucanase from *Acidothermus cellulolyticus* (Sigma) at 50 °C for 10 min, centrifuged at 10,000*g* at 4 °C for 10 min, and the supernatant was recovered for analysis of insoluble reaction products of CtPMO1.

### TLC

We applied TLC to analyze CtPMO1 products, applying samples to TLC on a Silica gel 60 F254 (Merck). We developed the plates using ethyl acetate:methanol:acetic acid:water (4:2:0.25:1, v/v) and visualized CtPMO1 products by heating them at 85 °C for 30 min with the chromogenic agent, which contained 4 mL phenylamine, 4 g diphenylamine, 30 mL 85% (w/w) phosphoric acid, and 200 mL acetone. Cellodextrin oligosaccharide mixture was used as markers.

### MALDI-TOF MS and MALDI-TOF MS/MS

We analyzed CtPMO1 using MALDI-TOF MS/MS. We applied samples to MALDI-TOF MS/MS on a 5800 MALDI-TOF/TOF analyzer (AB SCIEX) and analyzed them as described previously [6]. For MALDI-TOF MS measurements, we used an ionic preparation of 5-chloro-2-mecapto-benzothiazole (CMBT) and 2, 5-dihydroxybenzoic acid (DHB) as the matrix. 2 µL of the mixture of the samples and the matrix in a 1:1 ratio (v/v) was deposited on a target plate. The mass spectrometer was operated in the positive ion mode. MS data acquisition mass range was from *m/z* 500 to 1100. MS/MS data were acquired on the mass range of *m/z* range of 10–550. Fragmentation ion types were nominated as previously described [43].

### Analysis of CtPMO1 products oxidized by Br_2_

We used saturated bromine water (approximately 3%, w/v) to oxidize CtPMO1 products at 60 °C for 30 or 60 min and dried under a stream of nitrogen at 40 °C. Dried samples were then dissolved in water for MALDI-TOF MS analysis.

### NMR spectroscopy

CtPMO1 products were dissolved in DMSO-*d*_*6*_ solution and analyzed by NMR spectroscopy. NMR spectra were recorded at 25 °C on an Avance III 400 MHz instrument (Bruker), using TMS (*δ* = 0.00) as internal reference. One-dimensional spectra were acquired and processed using standard MestReNova software (Bruker).

### Site-directed mutagenesis

Site-directed mutagenesis of CtPMO1 was carried out according to the QuickChangeTM Site-Directed mutagenesis Kit (Stratagene, USA). The sequences of the primers used for CtPMO1 site-directed mutagenesis are shown in Additional file 1: Table S1. The PCR for mutation was performed with the following amplification program: 1 cycle at 95 °C for 2 min, 20 cycles composed of three steps for each cycle (95 °C for 20 s, 55 °C for 20 s, 72 °C for 2 min) followed by a final elongation step at 72 °C for 5 min. The mutated proteins were expressed and purified as the wild-type CtPMO1 protein.

### Homology modeling

Homology modeling of CtPMO1 was carried out using Swiss-Model server (http://www.swissmodel.expasy.org).

## Additional file

**Additional file 1: Figure S1.** SDS-PAGE of the purified Cu^2+^-CtPMO1 produced in *Pichia pastoris*. **Figure S2.** The N-terminal amino acid sequence analysis of CtPMO1 using LC-MS/MS. **Figure S3.** MALDI-TOF-MS/MS analysis of *m/z* 525 from MALDI-TOF-MS analysis. **Figure S4.** Types of fragmentation of CtPMO1 C4- and C6-oxidized products (m/z 525). **Figure S5.** ^1^H NMR spetra of CtPMO1 soluble reaction products with PASC as substrate in DMSO-*d*_*6*_. **Figure S6.** Sequence alignment of CtPMO1 and NCLPMO9C using ClastalW2. **Figure S7.** Homology model of the catalytic domain of CtPMO1 using SWISS-MODEL. **Figure S8.** Homology model of CtPMO1 binding with cellopentaose. **Figure S9.** Identification of the mutated CtPMO1 soluble reaction products oxidized by Br_2_ using with PASC as substrate MALDI-TOF-MS. **Table S1.** List of primers used for PCR of the CtPMO1 protein. **Table S2.** Fragmentation analysis of the peak of DP_3_-2 (m/z 525) according to Additional file 1: Figure S3, S4.

## Abbreviations

PMOs: polysaccharide monooxygenases
CtPMO1: a PMO from *Chaetomium thermophilum*
PASC: phosphoric acid-swollen cellulose
TFA: trifluoroacetic acid
AA: auxiliary activity
PCR: polymerase chain reaction
SDS-PAGE: sodium dodecyl sulfate polyacrylamide gel electrophoresis
LC–MS/MS: liquid chromatograph-tandem mass spectrometry
TLC: thin-layer chromatography
HPAEC-PAD: high-performance anion exchange chromatography with pulsed amperometric detection
DP: degree of polymerization
MALDI-TOF–MS/MS: matrix-assisted laser desorption/ionization-time-of-flight tandem mass spectrometry
LC–MS: liquid chromatograph–mass spectrometry
NMR: nuclear magnetic resonance
G1: glucose
G2: cellobiose
G3: cellotriose
G4: cellotetraose
G5: cellopentaose
G6: cellohexose
G7: celloheptaose
TMS: tetramethyl silane
