# Supplementary material for: Regioselectivity of oxidation by a polysaccharide monooxygenase from Chaetomium thermophilum
Source: Biotechnol Biofuels. 2018 Jun 5;11:155. doi: 10.1186/s13068-018-1156-2 (PMC5987470; doi:10.1186/s13068-018-1156-2)
Supplement: Supplementary file 1 — Additional file 1: Figure S1. SDS-PAGE of the purified Cu2+-CtPMO1 produced in Pichia pastoris. Figure S2. The N-terminal amino acid sequence analysis of CtPMO1 using LC-MS/MS. Figure S3. MALDI-TOF-MS/MS analysis of m/z 525 from MALDI-TOF-MS analysis. Figure S4. Types of fragmentation of CtPMO1 C4- and C6-oxidized products (m/z 525). Figure S5. 1H NMR spetra of CtPMO1 soluble reaction products with PASC as substrate in DMSO-d6. Figure S6. Sequence alignment of CtPMO1 and NCLPMO9C using ClastalW2. Figure S7. Homology model of the catalytic domain of CtPMO1 using SWISS-MODEL. Figure S8. Homology model of CtPMO1 binding with cellopentaose. Figure S9. Identification of the mutated CtPMO1 soluble reaction products oxidized by Br2 using with PASC as substrate MALDI-TOF-MS. Table S1. List of primers used for PCR of the CtPMO1 protein. Table S2. Fragmentation analysis of the peak of DP3-2 (m/z 525) according to Additional file 1: Figure S3, S4. [file 13068_2018_1156_MOESM1_ESM.doc]

**Additional information of this article can be found in the online version:**

**FIGURE S1. SDS-PAGE of the purified Cu^2+^-CtPMO1 produced in *Pichia pastoris*.**

Proteins were visualized by staining with Coomassie Brilliant Blue. M, protein marker (180, 130, 95, 72, 55, 43, 34, 26, 17, 10 kDa).

**M a**


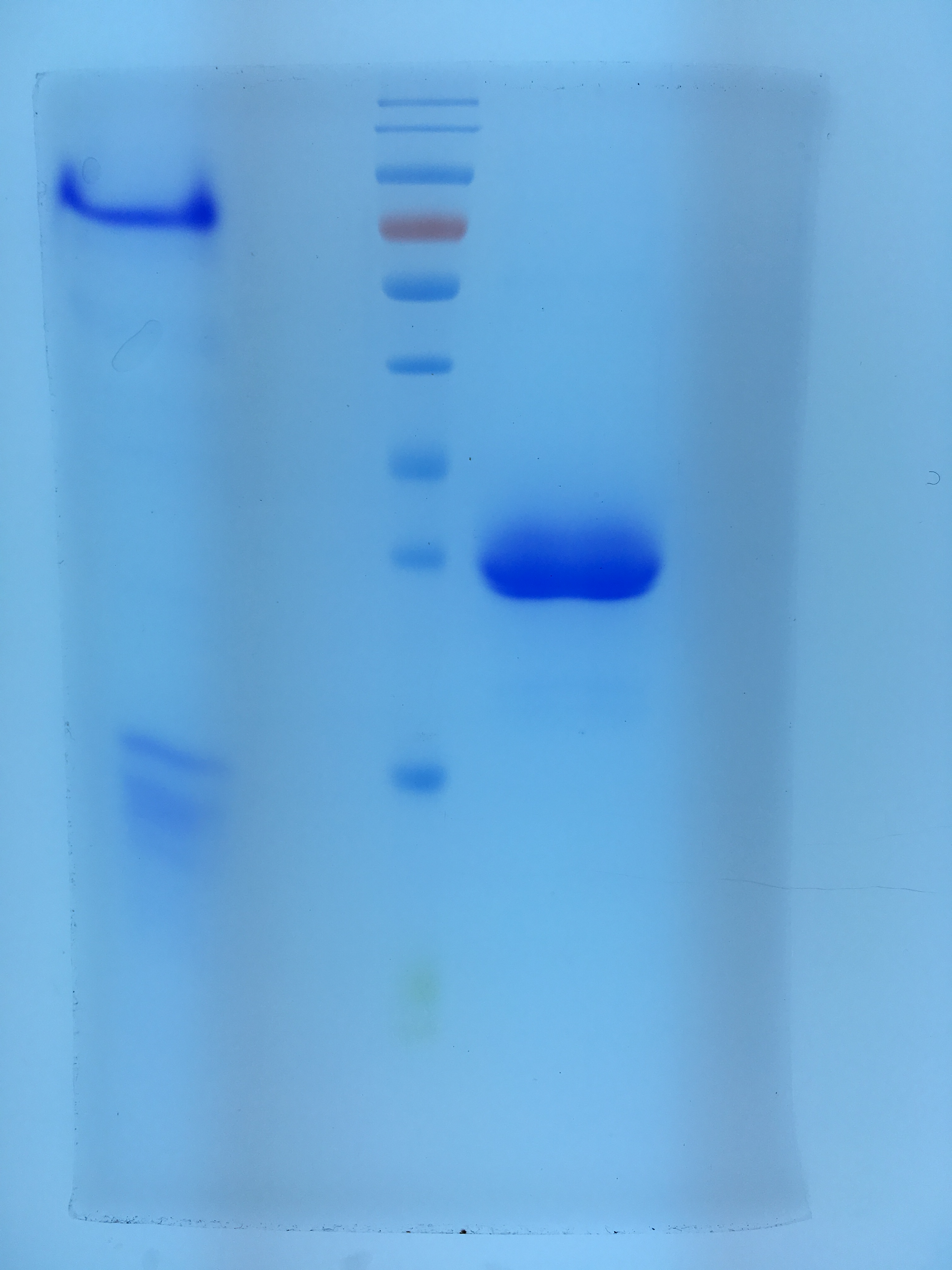


**FIGURE S2. The N-terminal amino acid sequence analysis of CtPMO1 using LC-MS/MS.**

MS analysis of the digested CtPMO1 protein with trypsin reveals a peak *m/z* 372.21. The *m/z* value is 1/2 of the molecular weight of the peptide HAIFQK (743.1 Da), indicating that the *m/z* 372.21 ion is doubly charged. Further MS/MS analysis shows that fragmentation *m/z* values of the *m/z* 372.21 ion are in agreement with the molecular weight of the corresponding fragmentations of the peptide HAIFQK (743.1 Da). These data indicate that the N-terminal amino acid sequence of CtPMO1 is HAIFQK.


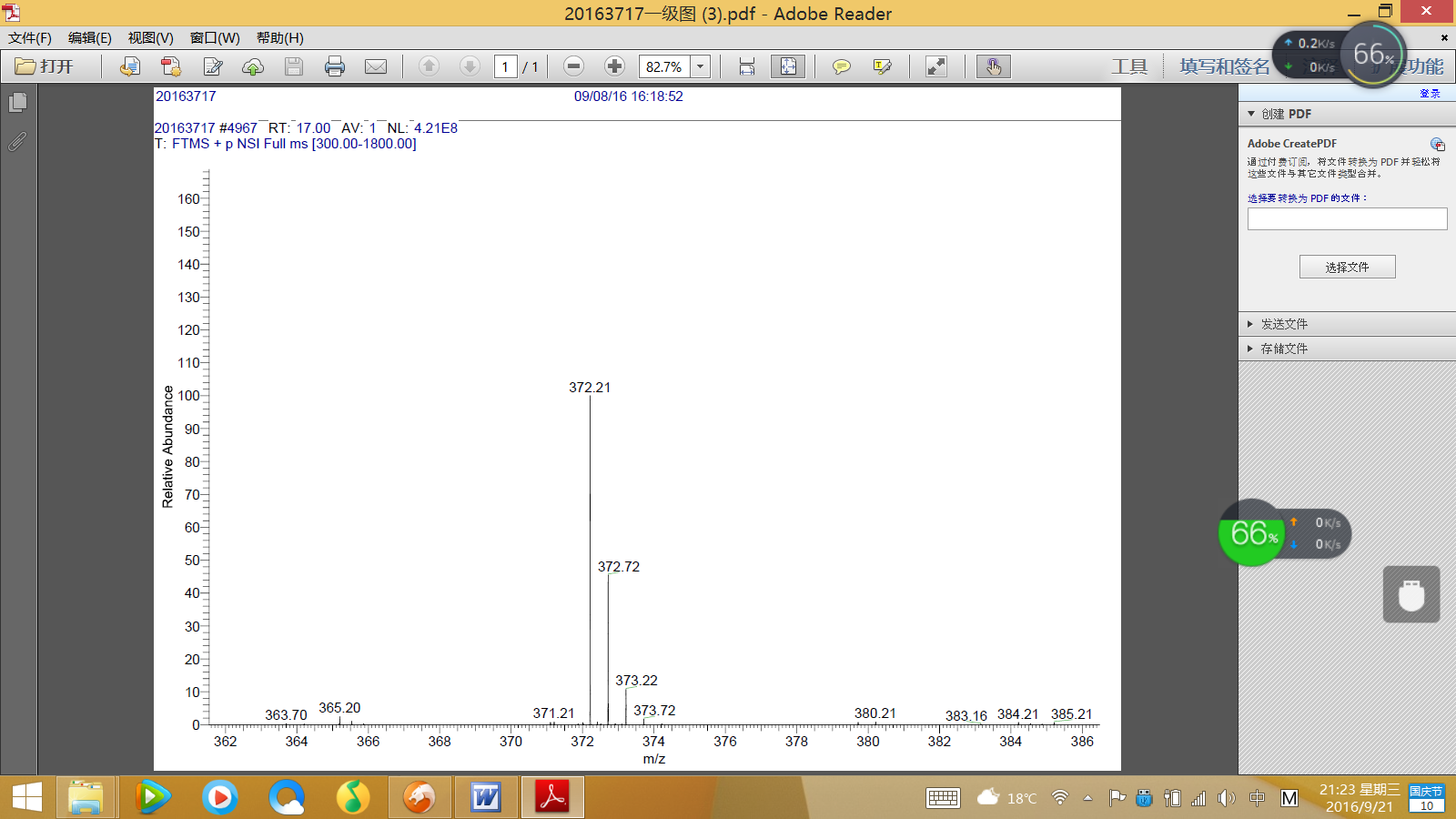


**MS**

Seq: HAIFQK

M: 743.1

**
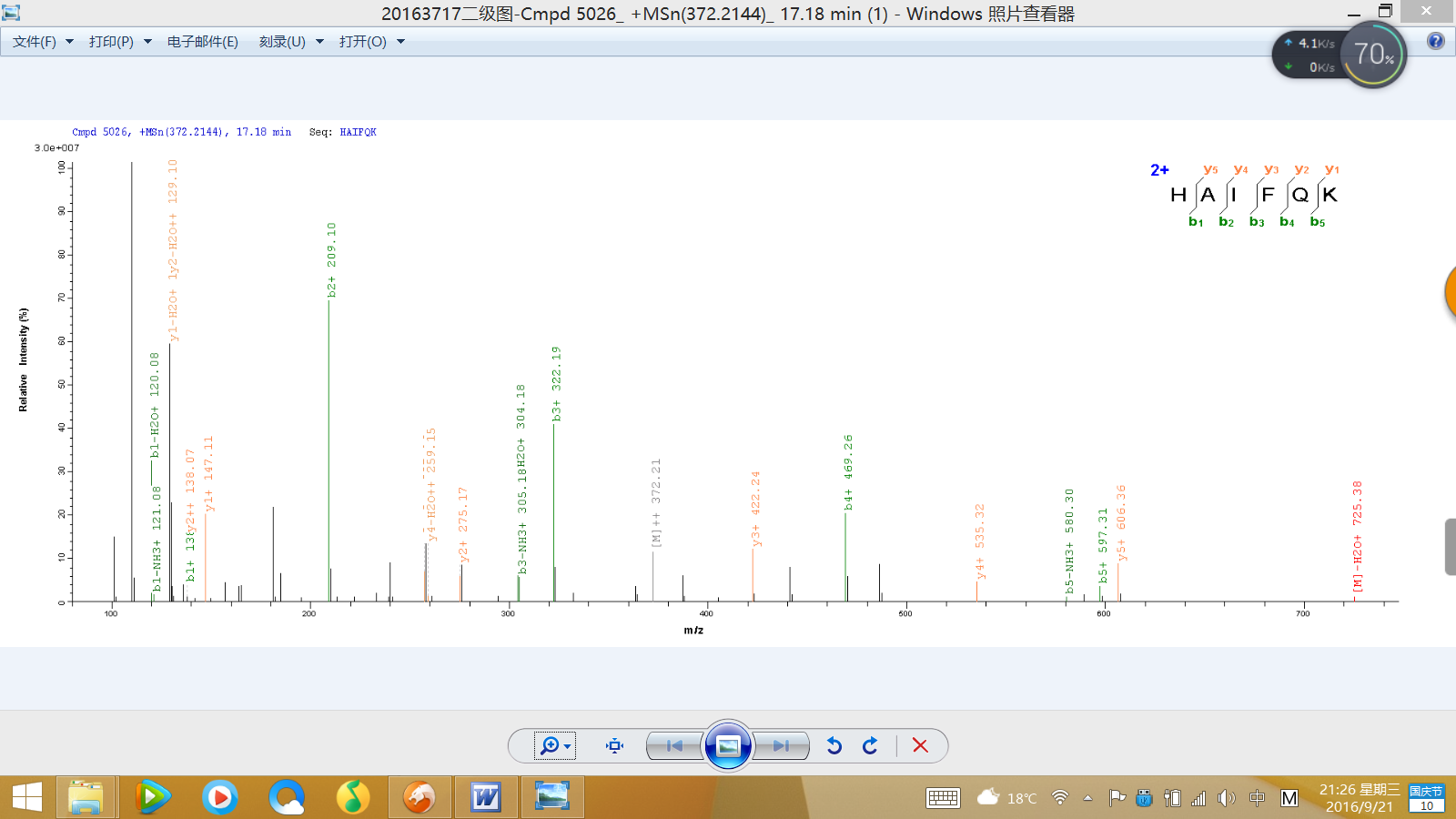
**

**MS/MS**

**FIGURE S3. MALDI-TOF-MS/MS analysis of *m/z* 525 from MALDI-TOF-MS analysis.**

We acquired MS/MS data on the mass range of *m/z* range of 10–550. We observed the various fragmentation ions of the main CtPMO1 C4 or C6 oxidized product (*m/z* 525). Table S2 shows the type of the fragmentation ions and the potential oxidized and non-oxidized products.

**

**

**m/z 300-550**

**

**

**m/z 10-300**

**FIGURE S4. Types of fragmentation of CtPMO1 C4- and C6-oxidized products (m/z 525).**

R represents -CH_2_OH or -CHO. There are three C-6 oxidized forms (*1*, *2*, *3*). *(1)*: R_1_, -CH_2_OH; R_2_, -CH_2_OH; R_3_, -CHO. *(2)*: R_1_, -CH_2_OH; R_2_, -CHO; R_3_, -CH_2_OH. *(3)*: R_1_, -CHO; R_2_, -CH_2_OH; R_3_, -CH_2_OH. Fragmentation ion types were nominated as previously described.

**
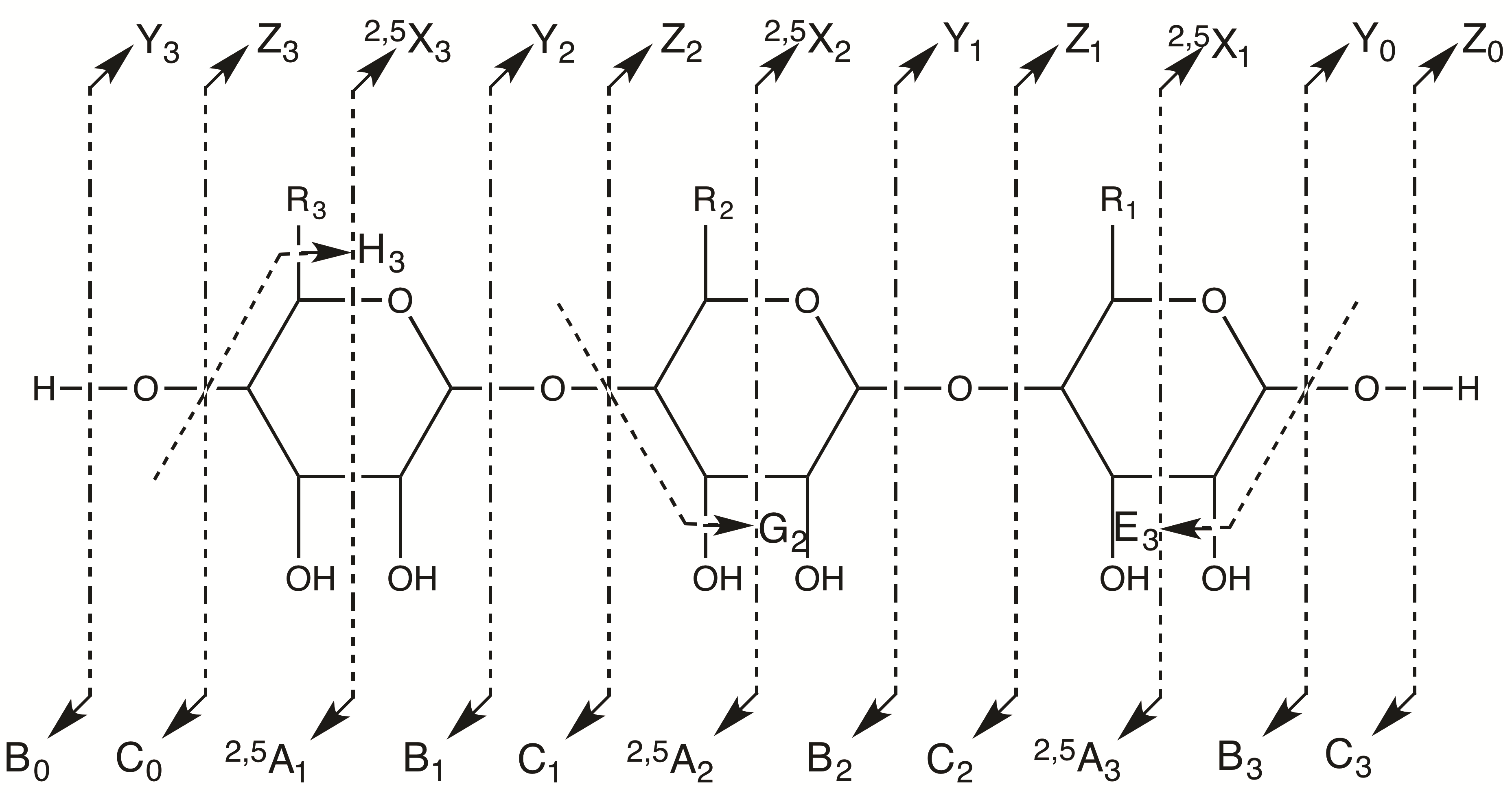
**

**FIGURE S5. ^1^H NMR spetra of CtPMO1 soluble reaction products with PASC as substrate in DMSO-*d_6_*.**

Soluble reaction products upon incubation of 0.5% PASC with CtPMO1 in 10 mM HAc-NH_4_Ac (pH 5.0) and 1 mM ascorbate at 50°C for 48 h. ^1^ H NMR spectrum of CtPMO1 soluble reaction products displayed an aldehyde proton signal at δ 8.39. The anomeric resonance at δ 8.39 is close to the anomeric resonances at δ 9.19 and 9.50 that are assigned to the aldehyde proton of two C6-oxidized galactose products (8).


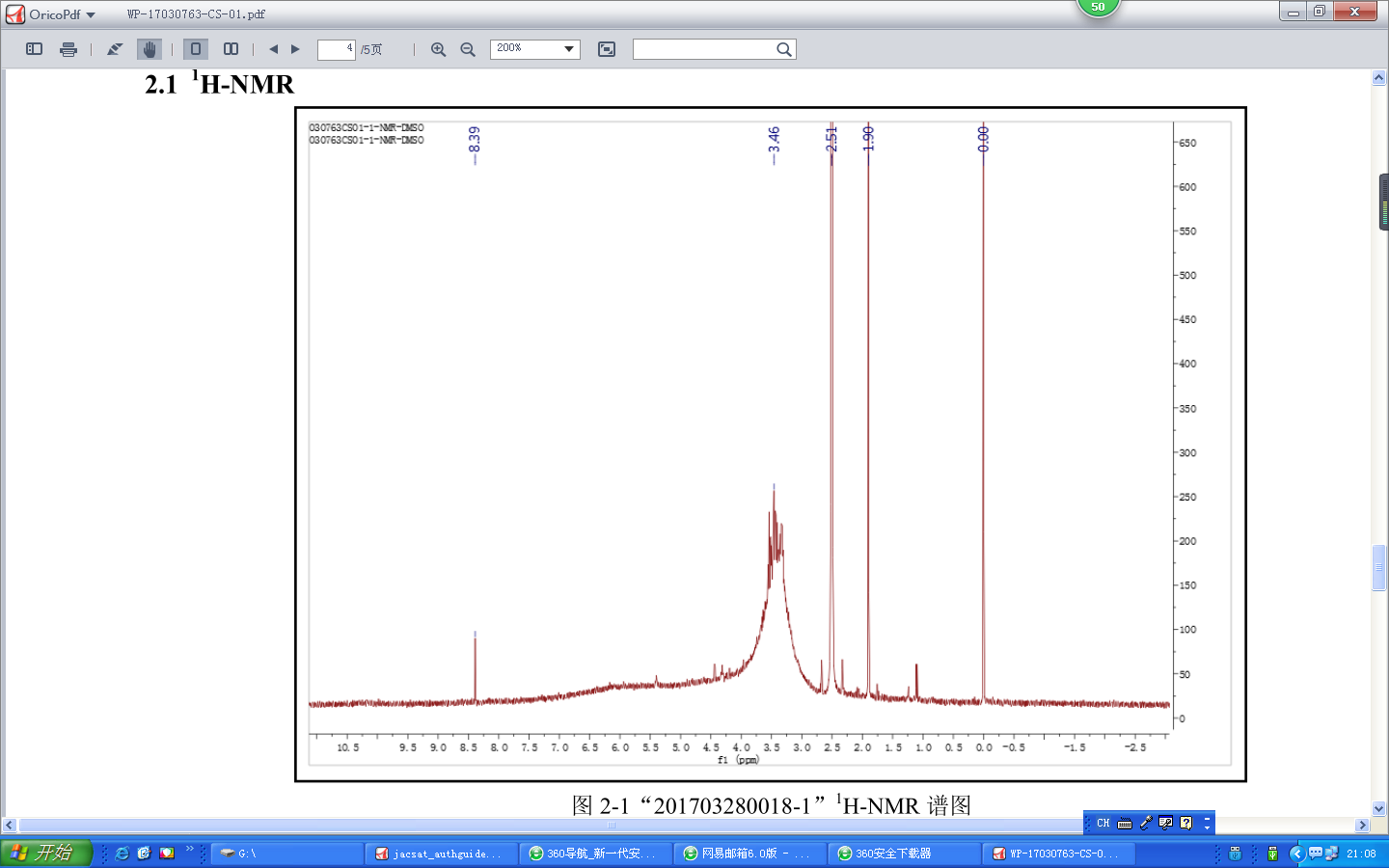


**DMSO-*d_6_*→**

**←HAc**

**←TMS**

**C6-hexodialdoses**

**FIGURE S6. Sequence alignment of CtPMO1 and NCLPMO9C using ClastalW2.**


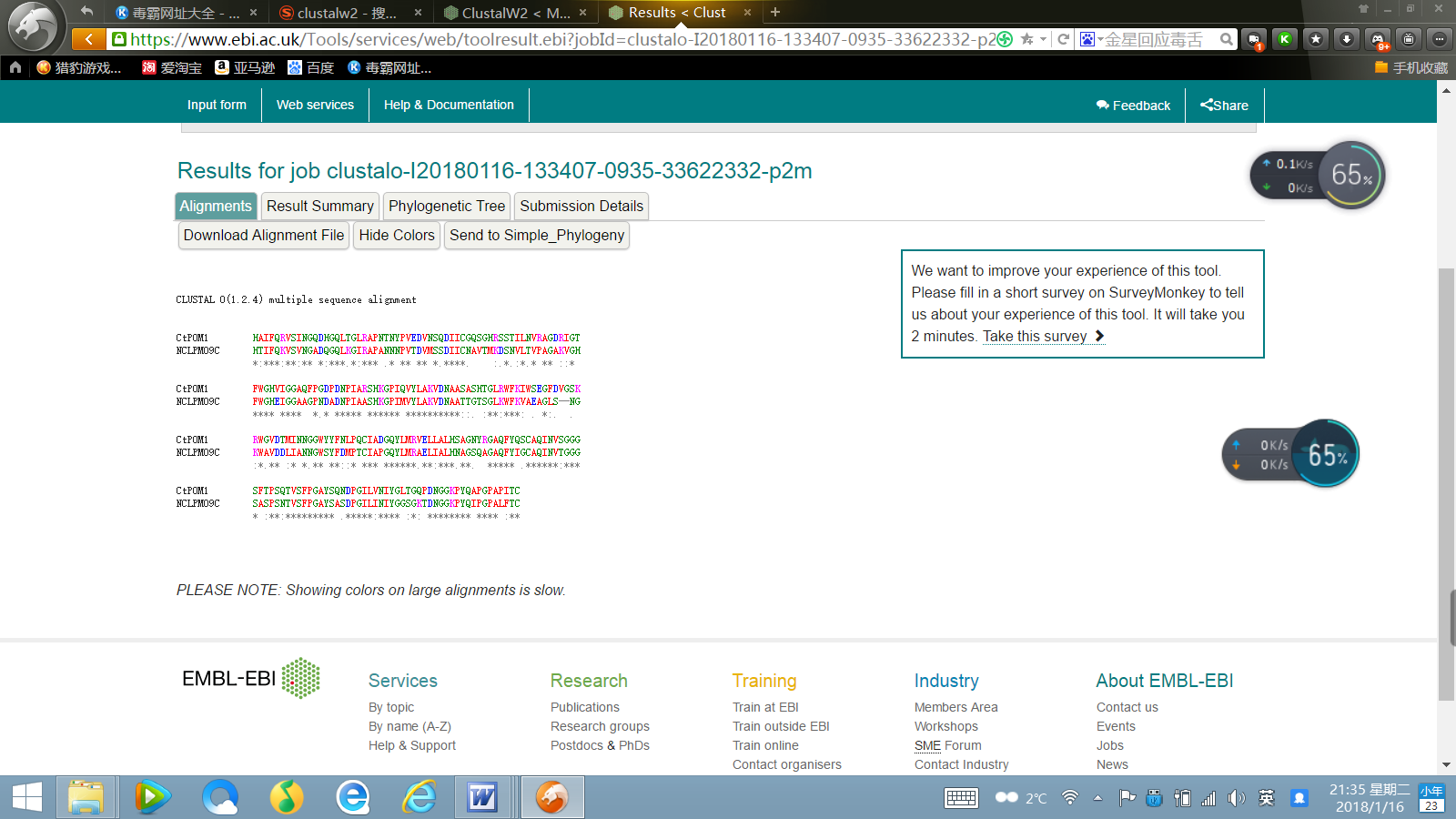


**FIGURE S7. Homology model of the catalytic domain of CtPMO1 using SWISS-MODEL.** The globally conserved residues adjacent to the copper are colored in yellow. The aromatic residues are colored in green. The copper ion is shown as an orange sphere.


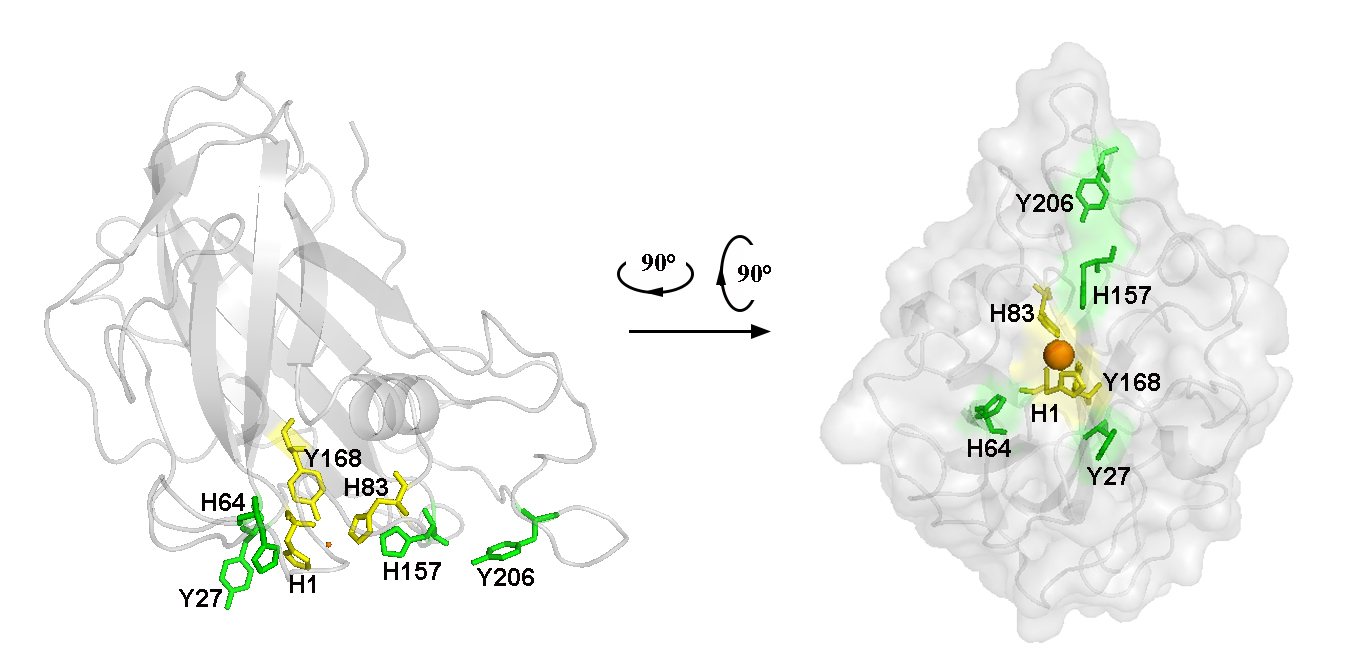


**FIGURE S8. Homology model of CtPMO1 binding with cellopentaose.** CtPMO1 model was aligned to LsAA9A:Cell5 (PDB ID: 5NLS) using PyMOL. The aromatic residues are colored in green. The copper ion is shown as an orange sphere. The carbon atoms of cellopentaose are colored in slate, and oxygen atoms of cellopentaose are colored in red.

**
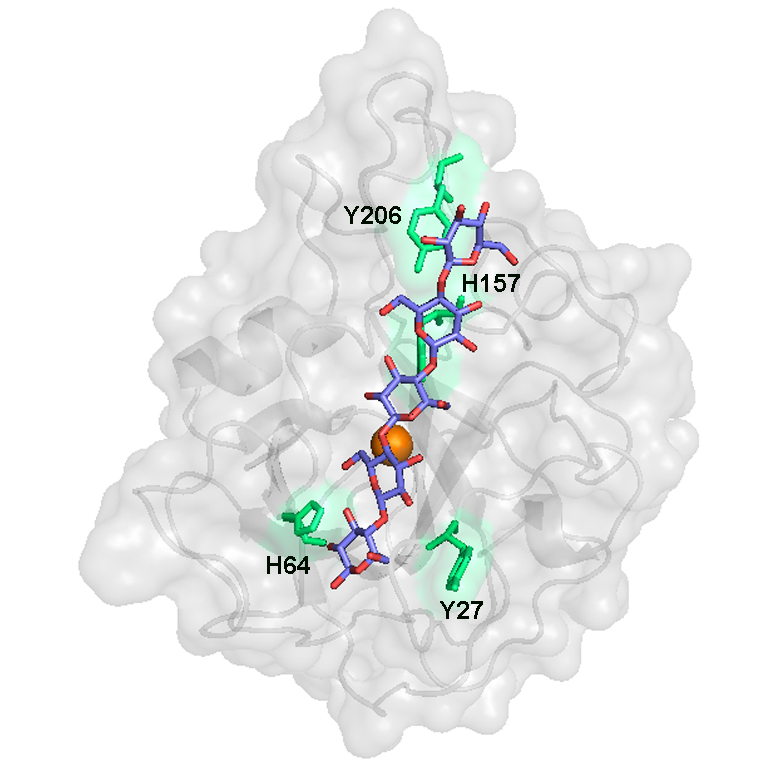
**

**FIGURE S9. Identification of the mutated CtPMO1 soluble reaction products oxidized by Br_2_ using with PASC as substrate MALDI-TOF-MS.**

Soluble reaction products upon incubation of 0.5% PASC with CtPMO1 in 10 mM HAc-NH_4_Ac (pH 5.0) and 1 mM ascorbate at 50°C for 48 h. C1-oxidized oligosaccharides (*m/z* +16), C4- and C1-oxidized oligosaccharides (*m/z* +14), C6- and C1-oxidized oligosaccharides (*m/z* +30), and C6-, C1- and C4-oxidized oligosaccharides (*m/z* +28).

**
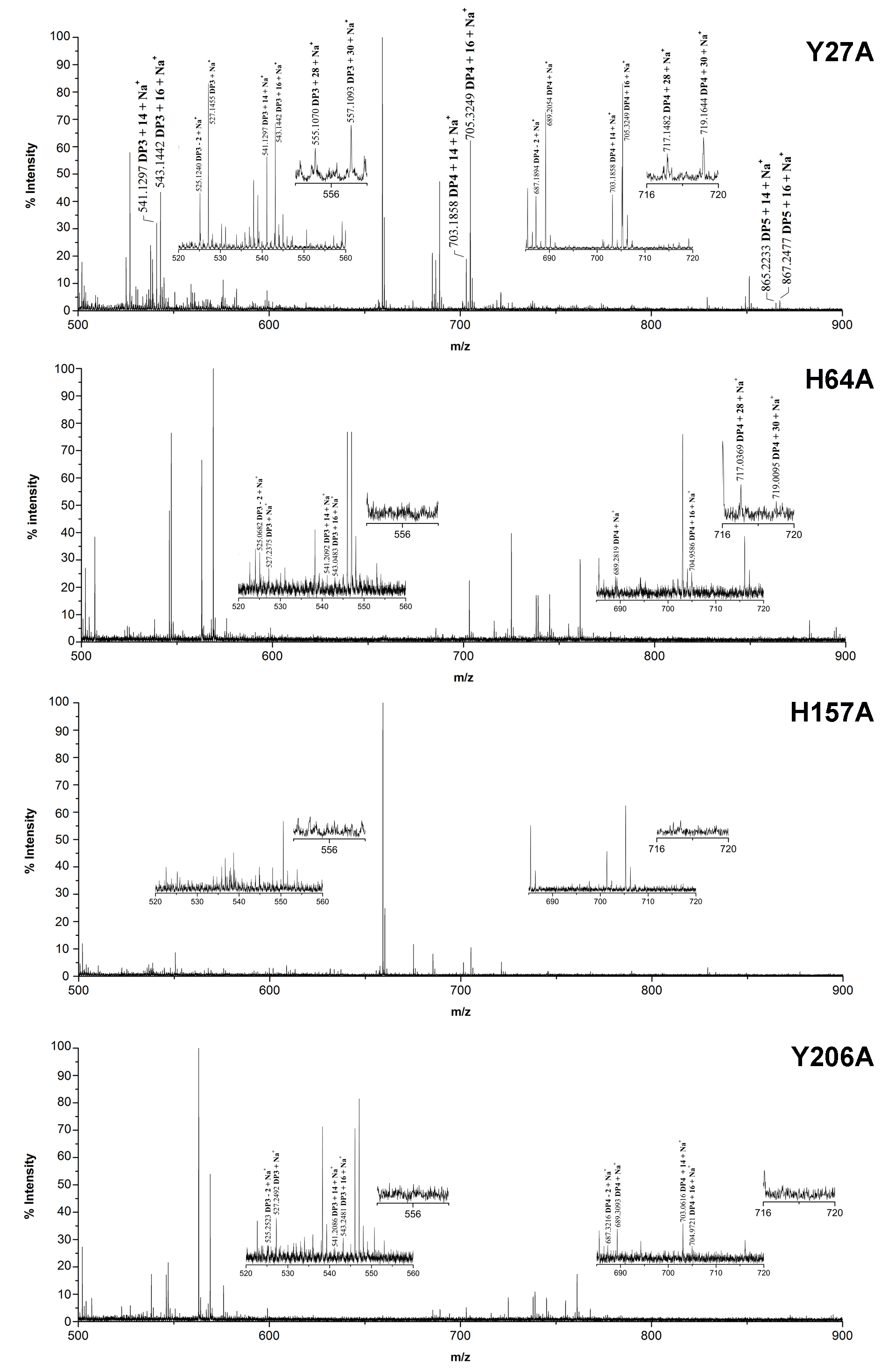
**

**Table S1 List of primers used for PCR of the CtPMO1 protein*.***

*Restriction sites for Xho I and Xba I are underlined.*

| Primers | Sequence | Purpose |
| --- | --- | --- |
| *Ct*PMO1-cF  *Ct*PMO1-cR  *Ct*PMO1-F  *Ct*PMO1-R  *Ct*PMO1-Y27AF  *Ct*PMO1-Y27AR  *Ct*PMO1-H64AF  *Ct*PMO1-H64AR  *Ct*PMO1-H157AF  *Ct*PMO1-H157AR  *Ct*PMO1-Y206AF  *Ct*PMO1-Y206AR | 5'-ATGAAACTTTCCTTG-3'  5'-GCACGTGATGGGAGC-3'  5'-AGGGGTATCTCTCGAGAAAAGACATGCCATCTTTCAG-3'  5'-GAGTTTTTGTTCTAGACCGCACGTGATGGGAGCCGG-3'  5'-GCGCCCAACACCAACGCCCCAGTTGAAG-3'  5'-GCGTTGGTGTTTGGGCGCACGCAGGCCGGTC-3'  5'-GGTACCTTTTGGGGCGCTGTCATTGGCG-3'  5'-GCGCCCCAAAAGGTACCAATTCTGTCAC-3'  5'-GAGCTGCTGGCTCTTGCCTCGGCTGGC-3'  5'-GCAAGAGCCAGCAGCTCGACACGCATG-3'  5'-CATCTTGGTCAATATCGCTGGCCTCACTG-3'  5'-GCGATATTGACCAAGATGCCCGGATCGTTC-3' | ORF cDNA of CtPMO1  ORF cDNA of CtPMO1  Expression of CtPMO1  Expression of CtPMO1  *Ct*PMO1 Y27A mutagenesis  *Ct*PMO1 Y27A mutagenesis  *Ct*PMO1 H64A mutagenesis  *Ct*PMO1 H64A mutagenesis  *Ct*PMO1 H157A mutagenesis  *Ct*PMO1 H157A mutagenesis  *Ct*PMO1 Y206A mutagenesis  *Ct*PMO1 Y206A mutagenesis |

**Table S2 Fragmentation analysis of the peak of DP_3_-2 (m/z 525) according to Fig. S3 and Fig. S4.**

| m/z | Potential fragmentation ion types | Potential products |
| --- | --- | --- |
| 521  513  503  492  490  488  479  477  467  466  465  454  437  424  423  421  419  399  397  381  369  367  365  349 | Y_3_ + H_2_O + H^+^ -2  C_3_ + H_2_O + H^+^ -2  ^3,4^X_3_+ Na^+^ +H_2_O -2  ^0,1^A_3_+ Na^+^ +H_2_O -2  Y_3_ + H^+^ -2  C_3_ + H^+^ -2  ^3,4^X_3_+ NH_4_^+^  ^3,4^X_3_+ NH_4_^+^-2  E_3_/G_3_+ NH_4_^+^  ^1,5^A_3_ -2 + Na^+^  H_3_ -2 + Na^+^  ^2,4^X_3_/^0,2^A_3_ + Na^+^  ^2,5^A_3_+ NH_4_^+^ + Na^+^- H^+^ -2  ^2,4^X_3_ + Na^+^ -2  ^0,2^A_3_+ Na^+^ -2  ^1,4^X_3_+ Na^+^+ NH_4_^+^- H^+^  ^3,5^A_3_+ Na^+^+H_2_O -2  ^0,2^X_3_+ Na^+^ + NH_4_^+^- H^+^  ^2,4^A_3_+ Na^+^ + NH_4_^+^- H^+^  ^2,4^A_3_+ Na^+^ +H_2_O -2  ^0,2^X_3_+ Na^+^ +H_2_O -2  ^3,5^A_3_+ Na^+^  ^3,5^A_3_+ Na^+^ -2  ^3,5^A_3_ + H^+^  ^3,5^A_3_ -2 + H^+^  Y_2_+ Na^+^ +H_2_O -2  C_2_+ Na^+^ +H_2_O -2  Z_2_/B_2_ + 2Na^+^ - H^+^  Z_2_ + 2Na^+^ - H^+^-2  B_2_ + 2Na^+^ - H^+^-2  C_2_/Y_2_ + Na^+^  ^3,4^X_2_ -2 + K^+^  ^0,1^A_2_ -2 + K^+^ | C6 oxidized products  C6 or C4 oxidized products  C6 oxidized products  C6 or C4 oxidized products  C6 oxidized products  C6 or C4 oxidized products  non-oxidized products  C6 oxidized products  non-oxidized products  C6 or C4 oxidized products  C6 oxidized products  non-oxidized products  C6 or C4 oxidized products  C6 oxidized products  C6 or C4 oxidized products  non-oxidized products  C6 or C4 oxidized products  non-oxidized products  non-oxidized products  C6 or C4 oxidized products  C6 oxidized products  non-oxidized products  C6 or C4 oxidized products  non-oxidized products  C6 or C4 oxidized products  C6 oxidized products  C6 or C4 oxidized products  non-oxidized products  C6 oxidized products  C6 or C4 oxidized products  non-oxidized products  C6 oxidized products  C6 or C4 oxidized products |

**Table S2 (continued)**

| m/z | Potential fragmentation ion types | Potential products |
| --- | --- | --- |
| 347  345  333  325  323  321  319  303  298  295  293  291  275  261  237  213    203  197  181  166  149  143  119  113  86  57  41  39  23 | Z_2_/B_2_ + Na^+^  Z_2_ + Na^+^-2  B_2_ + Na^+^-2  ^3,4^X_2_ -2 + Na^+^  ^0,1^A_2_ -2 + Na^+^  Z_2_/B_2_ + H^+^  Z_2_ + H^+^-2  B_2_ + H^+^-2  ^0,2^A_2_+ Na^+^+H_2_O -2  ^1,5^A_2_+ Na^+^  ^0,2^A_2_+ Na^+^ -2  ^2,4^X_2_+ Na^+^ -2  ^0,2^A_2_+ NH_4_^+^-2  ^2,4^X_2_+ NH_4_^+^ -2  H_2_ + H^+^  H_2_ -2 + H^+^  ^3,5^X_2_+ Na^+^  ^0,3^A_2_/^1,4^A_2_/^0,3^X_2_/^1,4^X_2_ + Na^+^  ^3,5^A_2_ +H_2_O -2+ Na^+^  ^0,2^X_2_+H_2_O -2+ Na^+^  ^3,5^A_2_ + H^+^  ^0,1^X_2_ -2 + Na^+^  ^3,4^A_2_ -2 + Na^+^  C_1_/Y_1_ + Na^+^  Y_1_ +H_2_O -2 + H^+^  C_1_ +H_2_O -2 + H^+^  C_1_/Y_1_ + H^+^  ^0,1^A_1_+ NH_4_^+^-2  ^3,4^X_1_+ NH_4_^+^-2  ^3,4^X_1_ -2+ H^+^  ^0,1^A_1_ -2 + H^+^  ^2,4^X_1_/^0,2^A_1_+ Na^+^  ^2,4^X_1_ -2+ H^+^  ^0,2^A_1_ -2 + H^+^  ^0,3^A_1_/^1,4^A_1_/^0,3^X_1_/^1,4^X_1_ + Na^+^  ^1,5^X_1_+ Na^+^ +NH_4_^+^ - H^+^  K^+^ +H_2_O  Na^+^ +H_2_O  K^+^  Na^+^ | non-oxidized products  C6 oxidized products  C6 or C4 oxidized products  C6 oxidized products  C6 or C4 oxidized products  non-oxidized products  C6 oxidized products  C6 or C4 oxidized products  C6 or C4 oxidized products  non-oxidized products  C6 or C4 oxidized products  C6 oxidized products  C6 or C4 oxidized products  C6 oxidized products  non-oxidized products  C6 oxidized products  non-oxidized products  non-oxidized products  C6 or C4 oxidized products  C6 oxidized products  non-oxidized products  C6 oxidized products  C6 or C4 oxidized products  non-oxidized products  C6 oxidized products  C6 or C4 oxidized products  non-oxidized products  C6 or C4 oxidized products  C6 oxidized products  C6 oxidized products  C6 or C4 oxidized products  non-oxidized products  C6 oxidized products  C6 or C4 oxidized products  non-oxidized products  non-oxidized products  hydrated metal ion  hydrated metal ion  metal ion  metal ion |
